# Transcriptomic Profiling of *Yersinia pseudotuberculosis* Reveals Reprogramming of the Crp Regulon by Temperature and Uncovers Crp as a Master Regulator of Small RNAs

**DOI:** 10.1371/journal.pgen.1005087

**Published:** 2015-03-27

**Authors:** Aaron M. Nuss, Ann Kathrin Heroven, Barbara Waldmann, Jan Reinkensmeier, Michael Jarek, Michael Beckstette, Petra Dersch

**Affiliations:** 1 Department of Molecular Infection Biology, Helmholtz Centre for Infection Research, Braunschweig, Germany; 2 Faculty of Technology and Center for Biotechnology (CeBiTec), Bielefeld University, Germany; 3 Department of Genome Analytics, Helmholtz Centre for Infection Research, Braunschweig, Germany; University of Würzburg, GERMANY

## Abstract

One hallmark of pathogenic yersiniae is their ability to rapidly adjust their life-style and pathogenesis upon host entry. In order to capture the range, magnitude and complexity of the underlying gene control mechanisms we used comparative RNA-seq-based transcriptomic profiling of the enteric pathogen *Y*. *pseudotuberculosis* under environmental and infection-relevant conditions. We identified 1151 individual transcription start sites, multiple riboswitch-like RNA elements, and a global set of antisense RNAs and previously unrecognized *trans*-acting RNAs. Taking advantage of these data, we revealed a temperature-induced and growth phase-dependent reprogramming of a large set of catabolic/energy production genes and uncovered the existence of a thermo-regulated ‘acetate switch’, which appear to prime the bacteria for growth in the digestive tract. To elucidate the regulatory architecture linking nutritional status to virulence we also refined the CRP regulon. We identified a massive remodelling of the CRP-controlled network in response to temperature and discovered CRP as a transcriptional master regulator of numerous conserved and newly identified non-coding RNAs which participate in this process. This finding highlights a novel level of complexity of the regulatory network in which the concerted action of transcriptional regulators and multiple non-coding RNAs under control of CRP adjusts the control of *Yersinia* fitness and virulence to the requirements of their environmental and virulent life-styles.

## Author Summary

Many bacterial pathogens cycle between environmental sources and mammalian hosts. Adaptation to the different natural habitats and host niches is achieved through complex regulatory networks which adjust synthesis of the large repertoire of crucial virulence factors and fitness determinants. To uncover underlying control circuits, we determined the first in-depth single-nucleotide resolution transcriptome of *Yersinia*. This revealed important novel genetic information, such as global locations of transcriptional start sites, non-coding RNAs, potential riboswitches and provided a set of virulence-relevant expression profiles, which constitute a valuable tool for the research community. The analysis further uncovered a temperature-induced global reprogramming of central metabolic functions, likely to support intestinal colonization of the pathogen. This is accompanied by a major reorganization of the CRP regulon, which involves a multitude of regulatory RNAs. The primary consequence is a fine-tuned, coordinated control of metabolism and virulence through a plethora of environmentally controlled regulatory RNAs allowing rapid adaptation and high flexibility during life-style changes.

## Introduction

Pathogenic bacteria have evolved a wide range of regulatory mechanisms to adjust their virulence strategies and biological fitness in response to fluctuating environmental conditions during the infection process. Expression of virulence-associated determinants is strictly and coordinately controlled by a set of transcriptional regulators and complex signal transduction cascades are responsible for sensing and integrating the environmental information into the virulence regulons [1–4]. In addition to the transcriptional control there has been increasing recognition that post-transcriptional mechanisms are used to fine-tune the management of virulence determinants and achieve a rapid and more refined control of the bacterial response [5–9].

Post-transcriptional regulation can be accomplished by (i) RNA thermosensors and riboswitches, i.e. regulatory elements of an mRNA, that undergo a structural alteration upon a thermal shift or binding of a small molecule [10–14], and (ii) antisense and *trans*-encoded small regulatory RNAs of which a subset bind to their target mRNAs by the help of the RNA chaperone Hfq [15]. The regulatory outcomes generally include alterations of the translation initiation efficiency or mRNA stability. In recent years a plethora of sensory and regulatory RNA elements has been identified in many human pathogens, including the genus *Yersinia* [16–20], and there is increasing evidence that they serve as crucial players in regulatory circuits adjusting cellular physiology, metabolism and virulence [6,21].

*Yersinia pseudotuberculosis* is a common foodborne pathogen that infects wild and domestic animals, as well as humans [22]. It is a very closely related ancestor of *Y*. *pestis*, the causative agent of plague, which has evolved from *Y*. *pseudotuberculosis* about 1,500 to 20,000 years ago [23,24]. Although both pathogens are genetically very similar (>97% nucleotide identity over 75% of the protein-coding genes), they differ significantly in their pathogenesis and exhibit very different infection and disease patterns [25,26]. In contrast to *Y*. *pestis*, *Y*. *pseudotuberculosis* causes a range of mild gut-associated diseases such as enteritis, watery diarrhea and mesenterial lymphadenitis, called yersiniosis. The intestinal diseases are usually self-limiting, but in rare cases *Y*. *pseudotuberculosis* can also trigger autoimmune responses [27,28].

Unlike *Y*. *pestis*, which typically colonizes fleas, *Y*. *pseudotuberculosis* is able to survive for considerable time periods in the soil and other environmental reservoirs [29]. Based on the distinct phases of their life-style, it is not surprising that sudden temperature and nutrient changes experienced upon entry from external/vector reservoirs into a warm-blooded host are the most important signals for *Y*. *pestis* and *Y*. *pseudotuberculosis* to trigger virulence gene expression and adjust their host survival program [30]. Thermal/nutrient shifts influence expression of multiple virulence-associated processes of *Yersinia*, e.g. host cell interaction, intracellular persistence, host immune defense, motility, and host-adapted metabolism. Recent work on individual virulence factors has illustrated that this is achieved by a joint interplay of transcriptional and post-transcriptional mechanisms, including *cis*-acting RNA elements and non-coding RNAs, that allow a more refined management of the bacterial response [6]. In this study, we used transcriptional profiling by RNA-seq to determine the transcriptional landscape of *Y*. *pseudotuberculosis* YPIII, identify thermal and nutrient control mechanisms on a global level, and elucidate the magnitude and regulatory architecture of the CRP regulon linking nutritional status to virulence. The CRP protein, a crucial global transcriptional regulator that interacts with cAMP and controls a plethora of genes in *Enterobacteriaceae* in response to the supply of glucose or other efficiently utilizable sugars [31,32], was previously shown to play a crucial role for the pathogenicity of *Yersinia* [33–36]. CRP modulates a large subset of virulence-relevant genes [34,37–39], and comparative metabolome and fluxome studies further revealed that absence of CRP strongly perturbs the carbon core metabolism at the level of the pyruvate-tricarboxylic acid cycle (TCA) node [34,39]. Transcriptional profiling of *Y*. *pseudotuberculosis* in this study further revealed a comprehensive remodelling of the CRP-controlled network in response to temperature and uncovered CRP as transcriptional master regulator of regulatory RNAs.

## Results and Discussion

### Differential and comparative RNA-seq of *Y. pseudotuberculosis* YPIII

*Y*. *pseudotuberculosis* strain YPIII is a widely distributed virulent isolate which played an important role for the analysis of *Yersinia* infection [34,35,40–45]. Many of its virulence factors have been characterized in detail and our knowledge of virulence-relevant gene regulation and networks was mainly derived from this strain. In order to obtain a comprehensive image of the transcriptome, we used rRNA-depleted total RNA of YPIII grown to exponential or stationary phase at 25°C or 37°C resembling alterations in temperatures and nutrient limitations encountered in the different life-styles.

To produce a detailed map and identify active transcriptional start sites (TSSs) at a single nucleotide resolution, we employed a global differential RNA-seq approach by comparing sequence reads from different strand-specific barcoded cDNA libraries [46]. Libraries denoted with +TAP were generated from RNA pools treated with tobacco acid pyrophosphatase (TAP) to allow 5’ adapter ligation to primary transcripts, and-TAP labelled libraries were generated from RNA pools, which were not treated with TAP (S1 Dataset; S1 Fig), similar to what has been described previously [47,48]. From each library between 2.3–12.9 million cDNA reads were generated and mapped to the YPIII genome sequence (NC_010465) and the pYV virulence plasmid (NC_006153).

**Fig 1:**
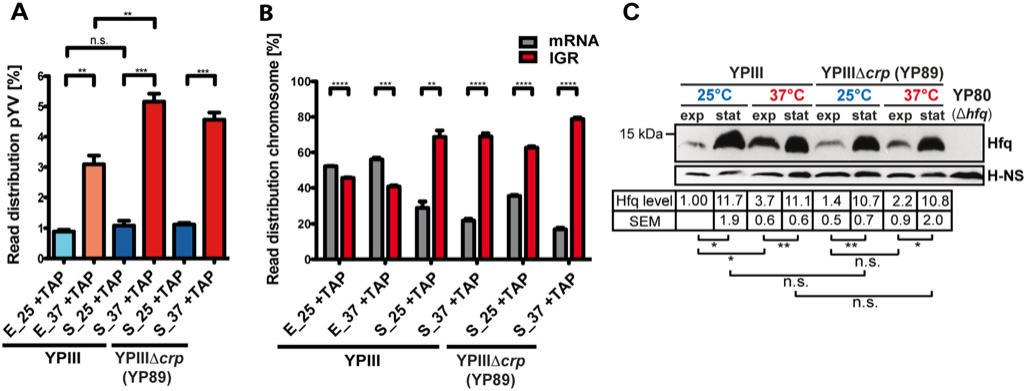
Global cDNA read count distribution and Hfq levels in *Y. pseudotuberculosis* during growth under environmental and infection-relevant conditions. (A) Percentage of uniquely mapped reads of the virulence plasmid pYV obtained by RNA-seq of *Y*. *pseudotuberculosis* YPIII and YPIIIΔ*crp* cDNA libraries. Libraries were generated from TAP-treated (+TAP) rRNA depleted RNA obtained from cultures grown to exponential (E) or stationary (S) growth phase at 25°C (25) and 37°C (37) (for detailed statistics see S1 Dataset). The data represent the mean ± SEM from three independent biological replicates and were analyzed with Student’s t-test. **: P<0,01; ***: P<0,001; n.s.: not significant. (B) Percentage of uniquely mapped reads to annotated ORFs (mRNA) or intergenic regions (IGR) of the YPIII genome. IGR reads do not include 5’-UTRs of mRNAs, rRNAs and tRNAs, and mostly represent *trans*-encoded sRNAs. The data represent the mean ± SEM from three independent biological replicates and were analyzed with Student’s t-test. **: P<0,01; ***: P<0,001; ****: P<0.0001. (C) Growth phase- and temperature-responsive expression of Hfq in *Y*. *pseudotuberculosis* YPIII and the isogenic *crp* deletion strain YP89. Equal amounts of whole cell extracts of YPIII or YP89 grown to exponential (exp) or stationary phase (stat) at 25°C or 37°C were separated by SDS-PAGE prior to Western blotting using polyclonal Hfq or H-NS antibodies (loading control). As a negative control YP80 (YPIII Δ*hfq*) was included. Relative protein amounts were quantified using ImageJ. The data represent the mean ± SEM from three independent experiments and were analyzed with Student’s t-test. *: P<0,05; **: P<0,01; n.s.: not significant.

Our RNA-seq approach confirmed earlier findings that expression of the virulence plasmid pYV-encoded genes is induced at 37°C [30,45,49–52], relative to the expression of the chromosome. In this context, enhanced expression of pYV at 37°C is pronounced for stationary phase cultures (Fig. 1A; S1 Dataset). In addition, we observed that the ratio between the intergenic region (IGR) and mRNA reads was considerably higher during stationary phase compared to exponential phase (Fig. 1B), indicating a pronounced expression of *trans*-encoded regulatory RNAs under nutrient-deprived growth conditions. Along with this observation we found that the intracellular level of Hfq, an RNA chaperone, which is often involved in the interaction of *trans*-encoded sRNAs with their target mRNAs [53], is substantially increased under nutrient starvation (Fig. 1C). This differs from *Escherichia coli* in which the abundance of Hfq varies only slightly and is maximal during log phase [54].

### Genome-wide analysis of TSSs

**Fig 2:**
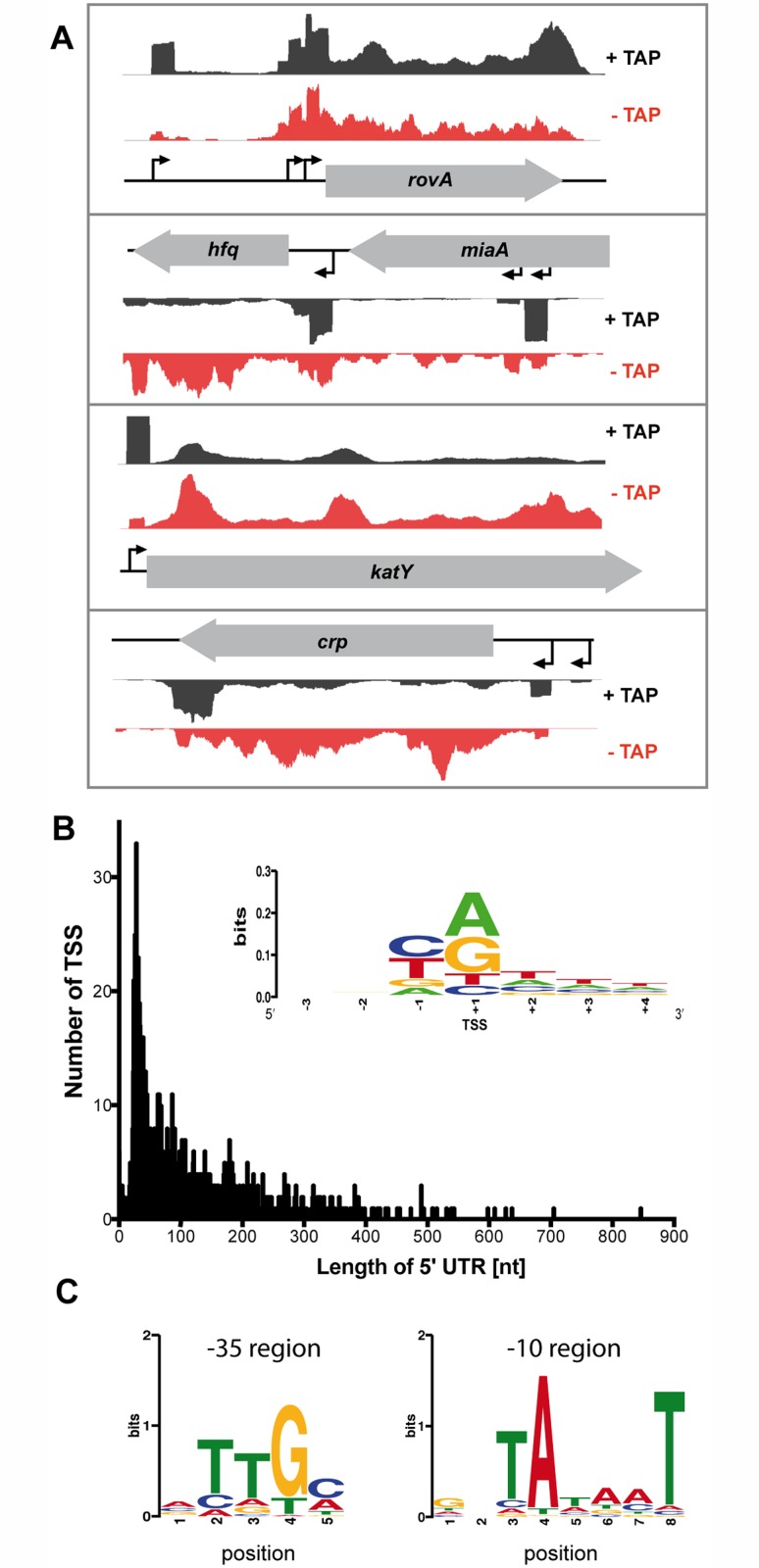
Global identification of mRNA transcriptional start sites (TSSs). (A) Visualization of RNA-seq (+/- TAP) based cDNA sequencing reads mapped to the YPIII *rovA*, *hfq*, *katY* and *crp* gene locus using the Artemis genome browser (Release 15.0.0). (B) The 5’-UTR repertoire. The distribution and frequency of the length of 5’-UTRs is given for all mRNAs of YPIII which start upstream of the annotated translational start site. More than 40% of all 5’-UTRs are 20–60 nt in length. (Inset) Sequence conservation at the TSSs. Sequence logo computed from 1151 unaligned TSS regions (TSS is located at position +1) showing nucleotide conservation around the TSSs. The initial nucleotide of transcripts (position +1) is dominated by purines while position -1 is dominated by pyrimidines. (C) Detected conserved sequence motifs in the -35 and -10 promotor region.

Annotation and comparison of the 5’-ends in the (+) vs (-) TAP cDNA libraries revealed a redistribution of the genome coverage profile towards an elevated sharp-edged 5’ flank, as illustrated for *rovA*, *hfq*, *katY* and *crp* (Fig. 2A). By a bioinformatic approach and other criteria (see Materials and Methods), we were able to identify 1151 individual TSSs in proximity to annotated open reading frames (ORFs) under all tested growth conditions, out of which 24 were mapped on the pYV virulence plasmid (S2 Dataset). In total, for 815 ORFs a single TSS was mapped, while 155 ORFs harbor at least one alternative TSS. The highest number of alternative TSSs was identified for *rpoD* and *gapA* for which transcription occurs from at least 5 different start sites (S2 Dataset).

To validate the TSSs, we compared predicted TSSs of the RNA-seq analysis with TSSs previously published for genes/operons of *Y*. *pseudotuberculosis* or close relatives and found that the vast majority of the identified TSSs start within 2 nt or are identical (S2 Dataset). For example, the identified TSS of *katY* was identical with the *katY* TSS identified in *Y*. *pestis* [55], and the two proximal TSSs of *rovA* matched perfectly with published start sites [56] (Fig. 2A; S2 Dataset). Interestingly, an additional *rovA* TSS was mapped in our approach, which is in perfect agreement with a third more proximal TSS identified within the *rovA* regulatory upstream region in *Y*. *enterocolitica* [57]. For *hfq* three individual TSSs were identified (Fig. 2A; S2 Dataset). Two TSSs reside within the coding region of the upstream gene *miaA*, similar to *hfq* from *E*. *coli* [58]. The most proximal *hfq* TSS at position -90 relative to the annotated *hfq* translational start site is in perfect agreement with a TSS recently determined for *hfq* of *Y*. *pestis* and *Y*. *pseudotuberculosis* [59], yet the TSS with the highest accumulation of cDNA reads under all tested conditions is the most distant one at position -509 (Fig. 2A; S2 Dataset). We further validated newly identified *Y*. *pseudotuberculosis* TSSs by 5’ RACE and found that 14 of the identified 16 TSSs in the RNA-seq approach matched with high accuracy (S2 Dataset).

To further strengthen our genome-wide analysis of TSSs, we investigated the nucleotide preference from position -3 to +4 surrounding the identified *Y*. *pseudotuberculosis* TSSs and generated a sequence logo using the WebLogo software (Fig. 2B) [60]. Our analysis revealed a significant dinucleotide preference at positions -1 and +1 while adjacent positions did not show any nucleotide preference. Similar to what has previously been reported for *E*. *coli*, *Salmonella enterica* serovar Typhimurium and *Klebsiella pneumoniae* [61,62], the nucleotide at position -1 is dominated by a pyrimidine base (37.01% C, 35.01% T), and transcription is preferentially initiated at a purine base (position +1; 42.75% A, 35.27% G) (Fig. 2B).

We further used our global TSS map to determine a motif for the -10 and -35 regions of *Y*. *pseudotuberculosis* promoters (Fig. 2C). To compute conserved sequence motifs, we performed *de-novo* motif discovery within the -10 region (position -15 to -3) and the -35 region (position -45 to -25) using MEME [63]. The detected conserved sequence motifs in the -35 (TTGc/a) and -10 promotor region (TAtaaT) are highly similar to those identified in other related *Enterobacteriaceae* [61,62].

### The 5’-UTR repertoire of *Y. pseudotuberculosis*

Our global TSS annotation further allowed us to define and analyze the untranslated regions (5’-UTRs) from the TSS to the start codon of the immediate downstream genes for putative *cis*-regulatory RNA elements, i.e. RNA riboswitches, known to control the translational efficiency of messengers [10–13]. Inspection of the 5’-UTRs of all 1151 defined transcriptional units of *Y*. *pseudotuberculosis* YPIII showed that the majority of 5’-UTRs is 20–40 nt in length (Fig. 2B; S2 Dataset) similar to other bacteria [64–66].

Notably, 155 genes are transcribed as mRNAs with 5’-UTRs longer than 200 nt, and a subset of 12 mRNAs had 5’-UTRs longer than 500 nt (S2 Dataset). Computational analysis using RibEx riboswitch explorer [67] predicted 4 known riboswitch-like elements (RLEs) among the long 5’-UTRs, e.g. cobalamin and FMN riboswitches, the *yybP*/*ykoY* element and the threonine operon leader (S2 Dataset). Moreover, a conserved RNA motif was predicted within the 217 nt 5’-UTR of *moaA*, encoding the molybdenum cofactor (Moco) biosynthesis protein A. Studies in *E*. *coli* demonstrated that the highly conserved RNA motif functions as Moco riboswitch [68]. RibEx predicted 17 additional RLEs; the respective candidates are mainly involved in metabolism and gene expression (S2 Dataset). For instance, the 5’-UTR of the *corA* mRNA of *Y*. *pseudotuberculosis*, encoding a magnesium/nickel/cobalt transporter (YPK_4002), harbors multiple RLEs. It likely represents a cation-responsive riboswitch similar to *mgtA* in *Salmonella*, encoding an Mg^2+^ transporter which is important for replication within macrophages [69,70].

We further report a small number of leaderless mRNAs with a 5’-UTR <10 nt (S2 Dataset). All 14 annotated leaderless mRNAs lack a classical ribosome-binding site and use an AUG as translation start codon, which was shown to be essential for stable ribosome-binding to leaderless transcripts [71]. Moreover, we identified 24 TSSs downstream of the annotated translational start site, indicating incorrect translational start site annotation. We compared the predicted sites with the start position of equivalent genes in other sequenced *Y*. *pseudotuberculosis* strains, including IP32953 and IP31758, and *Y*. *pestis* CO92 (S2 Dataset). For most of these genes, the annotated translational start site is located downstream of the translational start site given for YPIII in at least one of the other *Yersinia* genomes. One prominent example includes the virulence plasmid-encoded gene pYV0047 for the effector protein YopM. The annotation of the orthologous *yopM* gene on the PB1/+ virulence plasmid indicates a start codon that is located several nucleotides downstream of the TSS annotated for pYV (S2 Dataset). A putative ribosomal binding site (5’-AGGCA-3’) is located 6 nt upstream of the *yopM* translational start site annotated for the PB1/+ virulence plasmid, but it is lacking upstream of the translational start site annotated on pYV.

### The repertoire of *Y. pseudotuberculosis* non-coding RNAs

**Fig 3:**
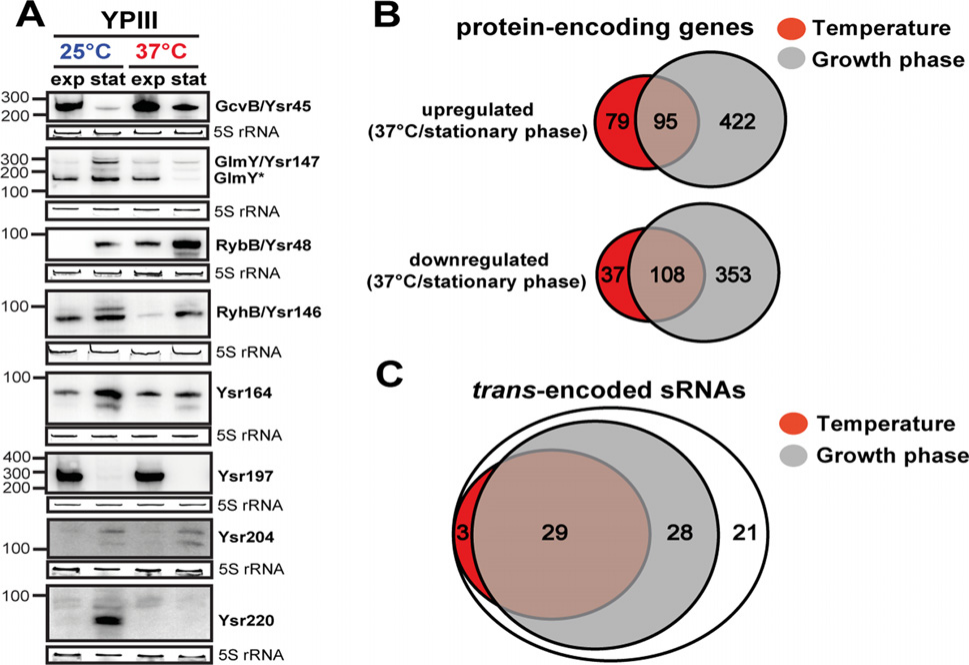
Temperature- and growth phase-responsive protein-encoding genes and *trans*-encoded RNAs. (A) Differential expression of *trans*-encoded sRNAs of YPIII shown by Northern blot analyses. RNA samples were prepared from bacteria grown to exponential (exp) or stationary phase (stat) at 25°C and 37°C. sRNAs were detected by specific radioactive labeled probes. 5S rRNA served as loading control. The size marker (nt) is indicated. Venn diagrams illustrating temperature and growth phase-regulated (B) protein-encoding genes and (C) *trans*-encoded sRNAs in *Y*. *pseudotuberculosis* YPIII. The regulons were obtained by comparative RNA-seq using DESeq from triplicate experiments (S3 and S4 Datasets). Protein- and *trans*-encoded RNAs which are differentially regulated by at least 4-fold (p-value ≤0.05) are included.

Several *trans*-encoded small RNAs (sRNAs) of different *Yersinia* strains have been recently discovered by deep sequencing of cDNA libraries enriched for small transcripts [16–20]. Our analysis of the *Y*. *pseudotuberculosis* YPIII transcriptome now allows us to investigate expression of sRNAs in relation to other transcripts under the different growth conditions. For identification of candidate regulatory RNAs a conservative strategy was used which defines non-coding RNAs as generally small transcripts (>40 nt and <500 nt) expressed from intergenic regions or the antisense strand of defined mRNAs. Besides the stable tRNAs and rRNAs also the predicted transfer messenger RNA (tmRNA, SsrA), RNase P (M1 RNA) and signal recognition particle (SRP RNA) were found to be expressed (S3 Dataset). We further identified 78 putative *trans*-encoded RNAs (S3 Dataset) and 80 putative antisense RNAs (S3 Dataset). All *trans*-encoded sRNA candidates are encoded on the YPIII chromosome. Conspicuously, 19 putative antisense RNAs are encoded on the virulence plasmid, a rather dense population compared to the low number of antisense RNAs identified for the YPIII chromosome. A comparison between *trans*-encoded sRNAs identified in this and previous studies [16–20] revealed a surprisingly low overlap of 38 sRNAs. We identified 42 new putative *trans*-encoded sRNAs for *Y*. *pseudotuberculosis* YPIII (S3 Dataset), whereas 143 previously described sRNAs were not detectable. This discrepancy is likely to be caused by low expression under the tested growth conditions, strain differences, RNA/library preparation protocols as well as bioinformatic criteria and pipelines applied for the RNA-seq approaches. Notably, several of the previously reported sRNAs were also classified as 5’-UTRs in our study, e.g. sR028, sR041, sR050, sR066 and sR070 which were identified in very close proximity to adjacent ORFs [20] (S2 Dataset). Potentially, those putative sRNAs are expressed as a premature transcript and result from processing of the 5’-UTR. This might account at least for sR066, for which a distinct short transcript was detected by Northern blotting [20]. In total, 36 of the non-coding RNA candidates were confirmed by Northern blotting (Figs. 3A, S2). This included 12 conserved sRNAs previously identified in other *Enterobacteriaceae*, 2 non-validated sRNAs (Ysr100 and Ysr103) and 1 validated sRNA (Ysr164) also identified in sRNA libraries of *Y*. *pestis* Kim6^+^ and *Y*. *pseudotuberculosis* IP32953 [16,18], 11 newly identified sRNAs and 10 antisense RNAs. Some of the validated antisense RNAs were identified as *trans*-encoded RNAs in *Y*. *pseudotuberculosis* IP32953 (Ysr93, Ysr15, Ysr114) [16]. Ysr15 and Ysr93 were grouped as antisense RNAs in this study, as they are encoded antisense to genes of hypothetical proteins of YPIII, which are not annotated in the IP32953 genome, and Ysr114 was found to largely overlap with the 3’-coding region of *trxB* (YPK_2687). How these antisense RNAs exert their function remains to be shown. For instance, the non-coding RNA McaS of *E*. *coli*, which regulates motility and biofilm formation is *trans*-acting, although it is encoded antisense to an annotated ORF [72].

### The temperature and growth phase-responsive regulons

To improve our understanding of the complexity of the regulatory networks controlling the multiple life-styles of yersiniae, we used our RNA-seq approach to identify coordinately regulated genes in response to temperature and nutrient limitation. Comparative RNA-seq analysis using DESeq [73] from triplicate experiments revealed a total of 324 ORFs of YPIII that were differentially regulated by at least 4-fold (p-value ≤0.05) in response to temperature (Fig. 3B; S4 Dataset). Growth phase had an even higher impact on global gene expression. Nearly 25% of the genome (983 genes) was differentially expressed (Fig. 3B; S4 Dataset). In accordance with previous data [30,45,52,74–76], many of the temperature-regulated genes encode well-known (i) regulators, effectors or components of the T3SS machinery on virulence plasmid pYV (of note, the components displayed a wide range of thermoregulation, of which the *lcrGVHyopBD*, *ypkA*, *yopE* and *yopH* transcripts were highly abundant even at 25°C), (ii) classical pathogenicity factors such as the adhesins AilA, PsaA, InvA, and the CNF_Y_ toxin, or (iii) other virulence-associated traits, e.g. urease, iron sequestration systems, motility/chemotaxis machinery and genes related to oxidative stress. The majority of the virulence-associated traits is also subject to growth phase control (S4 Dataset). This overlap is not solely restricted to the virulence-related attributes. Remarkably, 47% of the temperature-regulated genes encode metabolic functions, mainly enzymes of the carbon/energy metabolism, of which most are also subject to growth phase control (S4 Dataset). This observation and validity of our approach was confirmed by the strong correlation between comparative RNA-seq and qRT-PCR data of 24 randomly selected temperature- and/or growth phase-regulated genes encoding hypothetical proteins and proteins implicated in metabolism and virulence-relevant traits (S3 Fig). In summary, this indicated a clear link between both regulons in the control of *Yersinia* virulence.

**Fig 4:**
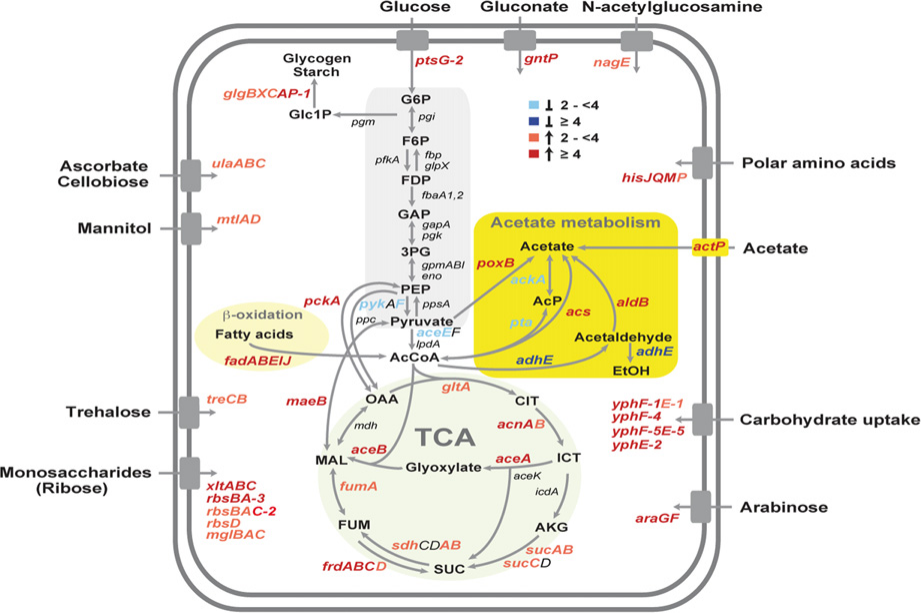
Temperature-dependent reprogramming of the primary metabolism of *Y. pseudotuberculosis*. Glycolysis, acetate metabolism, β-oxidation of fatty acids, the TCA cycle and transport systems are illustrated and the genes, designated according to the annotated YPIII genome, which encode equivalent enzymes of the presented pathways are given. Genes which are differentially expressed in response to temperature are colored. The blue color shows genes induced at 25°C (≥4, dark blue, ≥2 light blue), and the red color represents genes upregulated at 37°C (≥4, dark red, ≥2 light red). The values of the expression changes are listed in S4 Dataset.

Among the metabolic genes which are significantly upregulated at 37°C are genes encoding numerous sugar and amino acid transporters, enzymes involved in the catabolism of various carbohydrates and amino acids, β-oxidation of fatty acids, and energy production/conversion, indicating that terminal oxidation of many different substrates is favored at 37°C to allow rapid proliferation within the host (Fig. 4; S4 Dataset). Concomitant changes in oxidative catabolism, implicating a subset of these genes, were also found during temperature transition of the conditionally virulent *Y*. *pestis* strain KIM5 [77]. Most likely, these metabolic functions are part of the core regulon of thermoregulated genes in *Yersinia*.

One other striking observation which has not been observed in *Y*. *pestis*, is the existence of a thermoregulated ‘acetate switch’ (Fig. 4). This physiological switch occurs when bacteria transit from a program of rapid growth that produces acetate from acetogenic carbon sources, e.g. glucose, serine (dissimilation), to a program of slower growth facilitated by the import and utilization (assimilation) of acetate [78]. Similar to *E*. *coli* grown on high concentration of acetate, *Y*. *pseudotuberculosis* upregulates genes encoding enzymes with assimilation functions (PoxB, ActP, Acs), the glyoxylate bypass, the TCA cycle and gluconeogenesis (MaeB, PckA) at 37°C, while genes encoding PykF, AceEF, the Pta-AckA pathway and AdhE, converting acetyl-CoA to acetate or ethanol, were repressed (Fig. 4; S4 Dataset). A stronger induction of the acetate switch and the glyoxylate pathway was also observed under nutrient limiting condition during stationary phase, although expression of multiple enzymes of the TCA cycle remained unchanged (S4 Dataset). To support this observation, we compared the intracellular amount of two key metabolites of the acetate switch, acetate and acetyl-CoA, in *Y*. *pseudotuberculosis* grown at 25°C or 37°C, and found that the level of both metabolites was significantly reduced during growth at 37°C (S4 Fig).

Why flipping the switch in response to temperature? One reason could be that bacteria are primed for growth in the intestine. The mammalian intestinal tract is rich in short-chain fatty acids, of which acetate predominates. These volatile fatty acids are produced by the intestinal microbiota through consumption of available polysaccharides from diet or the host-secreted mucus and could constitute an important carbon/energy source for *Y*. *pseudotuberculosis* within the digestive tract [78,79]. Temperature-induced reprogramming to facilitate utilization of polysaccharide-derived degradation products is further supported by the fact that expression of several transport and catabolic genes of simple sugars are also thermally induced (Fig. 4). It is also possible that the acetate metabolism of *Yersinia* is influenced by the low availability of oxygen in the intestine. Studies with *E*. *coli* have shown that the absence of oxygen inhibits the expression of many TCA cycle enzymes which affects the pyruvate-acetate-acetyl-CoA flow [78].

Our global and quantitative RNA-seq approach further revealed a strikingly large number of growth phase- and/or temperature responsive *trans*-encoded sRNAs (74%; Fig. 3A, C; S3 and S4 Datasets). The vast majority (86%) of the growth phase-controlled sRNAs are present at higher abundance during stationary phase of which about half also respond to temperature (Fig. 3A, C; S3 Dataset). Among them are most conserved sRNAs, e.g. RybB and many novel sRNAs, e.g. Ysr220. However, there are also prominent exceptions to this trend, e.g. GcvB and Ysr197, which are strongly repressed during stationary phase (Fig. 3A; S3 Dataset). The large number of temperature- and growth phase-responsive sRNAs is likely to contribute an important additional control layer to adjust and fine-tune the pathogen’s fitness and virulence in the response to the dramatic changes associated with the transmission of yersiniae between heterothermic environments and homeothermic mammals. The physiological relevance of these sRNAs for pathogenesis is further supported by a previous study, reporting the importance of the RNA chaperone Hfq for the virulence of *Y*. *pseudotuberculosis* [80].

### Reprogramming of the CRP regulon by temperature

One crucial global regulator previously shown to link the nutritional status of *Yersinia* to virulence is CRP. CRP controls the transcription of multiple genes and operons in *Enterobacteriaceae* in response to certain carbon sources (in particular simple sugars), which are scarse during stationary phase. Loss of CRP was found to affect transcription of many genes involved in the primary metabolism and pathogenicity in *Y*. *pestis*, *Y*. *enterocolitica* and *Y*. *pseudotuberculosis* and led to a strong attenuation of their virulence [33,34,36–38,81]. This tight link between catabolite repression and virulence motivated us to include a *crp* deficient mutant of *Y*. *pseudotuberculosis* YPIII (YP89) into our transcriptomic approach to (i) compare the influence of CRP at temperatures mimicking environmental/vector or mammalian host niches, and (ii) address global impact of CRP on the expression of sRNAs.

Our analysis revealed 741 CRP-dependent genes (S5 Dataset) of which 228 were previously identified by a microarray approach (62% overlap) which addressed expression solely at 25°C during late stationary phase [34]. Strikingly, only a small proportion of genes (142 genes, 19%) is shared between the CRP regulons determined at 25°C or 37°C, as exemplified by the *acs* and *mdh* genes, while the majority varies significantly. For instance, *aceBAK* transcript abundance is negatively affected by CRP at 37°C, but not at 25°C (S5 Dataset; S5 Fig). This is also reflected by the finding that the composition of temperature-regulated genes is dramatically altered in the *crp* mutant. Loss of *crp* had only a minor influence on the expression of the pYV-encoded virulence genes, whereas the majority of the temperature-dependent chromosomal genes (191 genes, 75%), including numerous metabolic, virulence and stress adaptation genes, did not respond to temperature in the absence of CRP (S6 Dataset). In contrast, 247 genes are solely temperature-regulated in the *crp* deletion strain, the majority of which is negatively affected by CRP. In summary, our data reveal a profound reprogramming of the CRP regulon by temperature (S6 Dataset). How this is accomplished is unclear, but the large number of transcriptional regulators which are members of the two distinct CRP regulons (S5 Dataset; S5 Fig) indicates that the switch is supported by CRP-dependent, thermo-controlled regulators such as RovA [34,82,83]. This global virulence regulator is only expressed and positively controlled by CRP at 25°C, but not at 37°C due to the fact that RovA is a protein thermometer which is partially defolded and rapidly degraded at 37°C [82].

### CRP, a master regulator of sRNAs in *Y. pseudotuberculosis*

**Fig 5:**
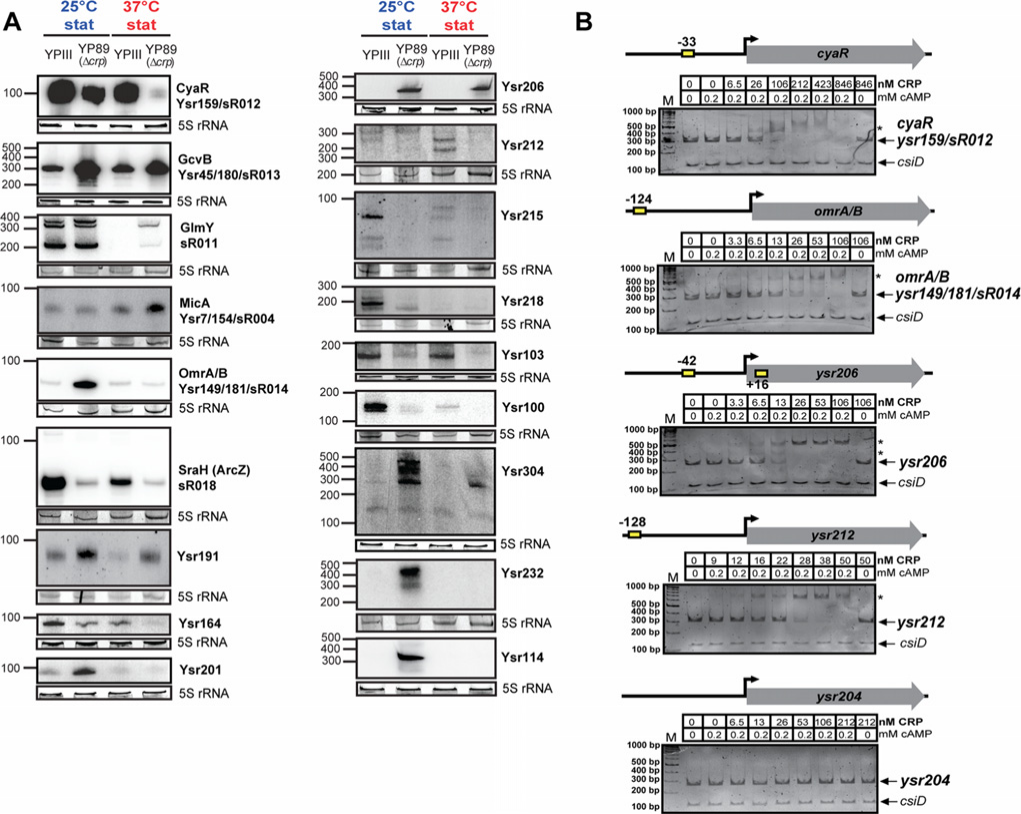
CRP-dependent non-coding RNAs of *Y. pseudotuberculosis*. (A) Northern blot analyses of selected CRP-regulated non-coding RNAs. RNA samples were prepared from YPIII and YP89 (YPIII Δ*crp*) bacteria grown to stationary growth phase (stat) at 25°C and 37°C. 5S rRNA served as loading control. The size marker (nt) is indicated. (B) Interaction of CRP with the regulatory regions of selected CRP-regulated sRNA genes. Individual DNA fragments with the predicted CRP-binding site(s) (yellow boxes; S3 Dataset) used for electrophoretic mobility shift assays are illustrated. An individual sRNA promoter fragment (*ysr204*) for which no CRP-binding site was predicted was included as negative control. Respective DNA fragments were incubated with increasing concentrations of CRP and 0.2 mM cAMP. As a negative control, cAMP was omitted in samples with the highest CRP concentration (right lane). The CRP-DNA complexes were separated on 4% polyacrylamide gels. The position of specific higher molecular weight complexes is marked with an asterisk. A molecular weight standard (M) was loaded, and the corresponding molecular weights are indicated. A *csiD* PCR fragment amplified from *E*. *coli* served as an internal negative control.

The accumulation of regulatory RNAs during stationary phase suggested that they might also be part of the CRP regulon. Our investigation of transcriptomic changes revealed 53 *trans*-encoded RNAs of *Y*. *pseudotuberculosis* (65%) which are up- or down-regulated in the absence of *crp* at 25°C and/or 37°C (S3 and S5 Datasets). Many conserved *trans*-encoded RNAs, but also multiple newly identified sRNAs were identified as members of the CRP regulon, and their intracellular level is strongly affected by temperature. CRP-dependent regulation of all 15 randomly selected *trans*-encoded sRNAs was confirmed by Northern blotting (Fig. 5A). Furthermore, we validated one newly identified *trans*-encoded RNA Ysr206, as well as two antisense RNAs Ysr232 and Ysr114, which were exclusively detected in the *crp* mutant (Fig. 5A). Both antisense RNAs constitute the most abundant antisense RNAs identified by our approach (S3 Dataset). The huge subset of CRP-regulated non-coding RNAs strongly indicates that CRP is a master regulator of regulatory RNAs in *Y*. *pseudotuberculosis*. Recent identification of four CRP-dependent riboregulators in *Y*. *pestis* (CyaR, sR065, sR066, sR084 encoded on pPCP [20]) further indicate that this might also account for other *Yersinia* species. Furthermore, CRP-mediated control seems to account for the accumulation of many sRNAs during stationary phase, and it also explains downregulation of a subset of sRNAs (e.g. GcvB), which are negatively affected by the regulator (Figs. 3A, 5A).

### CRP interacts with the regulatory upstream region of multiple *Yersinia* sRNAs

A manual search and a string-based prediction of CRP binding motifs (TGTGA-N_6_-TCACA) within the regulatory region of all 54 CRP-dependent sRNA genes identified one or more putative CRP binding sites within 18 of these sRNA genes (S3 Dataset). Direct and specific CRP binding in a cAMP-dependent manner was found for *cyaR*, *omrA*, *sraH*, Ysr100, 191, 206, 212, 215, 218, 226, and a manually identified sRNA, Ysr304 (Figs. 5B, S6). Cooperative interactions were observed with the regulatory region of Ysr206 and Ysr226, for which more than one CRP binding site was predicted (Figs. 5B, S6; S3 Dataset). The validity of our data was reinforced by the fact that CRP did not interact with the regulatory regions of *ysr204*, *rybB* and *ysr232* for which no CRP binding site was predicted (Figs. 5B, S6). To confirm these results we also selected two CRP-dependent promoter regions of sRNA genes (sR018 and *ysr212*), which have previously been identified to be CRP-dependent (Figs. 5B, S6; S3 Dataset) and performed DNase I footprinting experiments. Incubation of the fragments with increasing CRP concentrations revealed that CRP binds specifically to the promoter regions at the predicted CRP-binding sites (S7 Fig). This demonstrates that a large proportion of the *Yersinia* sRNAs are under direct control of CRP. Catabolite repression of the remaining sRNAs might be mediated indirectly by other transcriptional regulators (e.g. RpoN for GlmY [84], and RpoE for RybB [85,86] identified as part of the CRP regulon; S5 Dataset; S5 Fig).

### Regulation of CRP in response to growth phase and temperature

**Fig 6:**
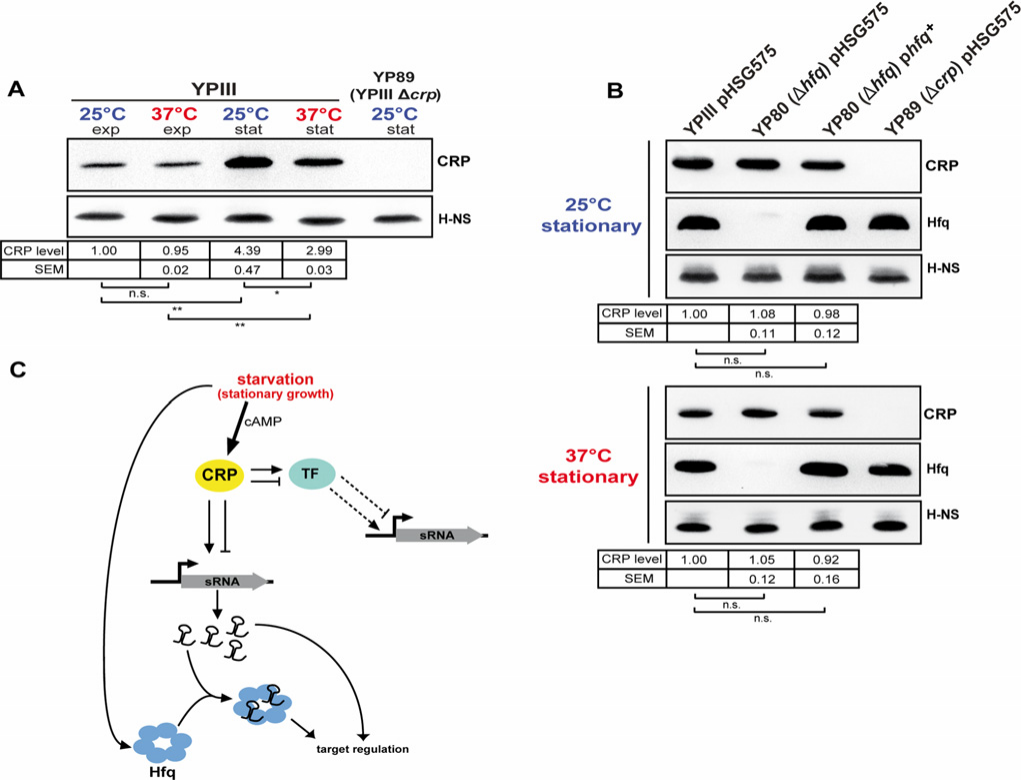
CRP, a growth phase and temperature-responsive master regulator in *Y. pseudotuberculosis*. (A) CRP protein levels in response to growth phase and temperature. Equal amounts of whole cell extracts of YPIII grown to exponential (exp) or stationary phase (stat) at 25°C and 37°C were separated by SDS-PAGE prior to Western blotting using polyclonal CRP antibodies. H-NS served as loading control. YP89 (YPIII Δ*crp*) was included as negative control. Relative protein amounts were quantified using ImageJ. The data represent the mean ± SEM from three independent experiments analyzed with Student’s t-test. *: P<0,05; **: P<0,01; n.s.: not significant. (B) Effect of the RNA chaperone Hfq on CRP. Equal amounts of whole cell extracts of the wild-type strain YPIII, the isogenic *hfq* mutant (YP80) and the complemented *hfq* mutant (YPIII pBW131 (p*hfq*+)) grown to stationary phase at 25°C and 37°C were separated by SDS-PAGE prior to Western blotting using polyclonal CRP or Hfq antibodies. Plasmid pHSG575 served as the empty vector control. YP89 (YPIII Δ*crp*) was included as negative control. H-NS served as loading control. Relative protein amounts were quantified using ImageJ. The data represent the mean ± SEM from three independent experiments analyzed with Student’s t-test. n.s.: not significant. (C) Current model of growth phase- and temperature-dependent sRNA expression involving the transcriptional regulator CRP and the RNA chaperone Hfq. During stationary growth when glucose is limiting intracellular CRP and cAMP levels increase, leading to accumulation of active CRP protein. Active CRP induces the expression of numerous sRNAs by direct binding to the regulatory region of the sRNA gene, thereby stimulating or repressing transcription. Further, expression of those sRNAs which are not directly regulated by CRP might involve one or more of the CRP-dependent transcription factors (TF), whose expression is differently affected by temperature. During stationary growth the RNA chaperone Hfq accumulates, similar to CRP, and affects sRNA abundance on the post-transcriptional level.

The tight link between catabolite repression and thermal control in *Yersinia*, in particular when one considers the expression of non-coding RNAs, suggested that synthesis and/or activity of CRP itself might be subjected to thermal and growth phase control. Indeed, we found that expression of the cAMP-producing adenylate cyclase gene *cyaA* was 2.8-fold increased during stationary phase (S4 Dataset). Moreover, CRP levels are strongly increased during stationary phase, whereby the amount of the global regulator is slightly but significantly higher at 25°C compared to 37°C (Fig. 6A). Interestingly, the *crp* transcript levels quantified by RNA-seq do not increase during transition from exponential to stationary growth (S4 Dataset), indicating that environmental control in response to growth phase occurs on the post-transcriptional level. Post-transcriptional control of CRP was also reported in *Y*. *pestis* CO92 in the context of the control of the plasminogen activator protease Pla [36]. In *Y*. *pestis*, loss of the RNA chaperone Hfq was found to decrease CRP levels. The molecular mechanism is not yet known, but it was found to involve the *crp* 5’-UTR [36]. We further tested whether loss of Hfq has also an influence on the amount of CRP in *Y*. *pseudotuberculosis* (Fig. 6B). However, no significant difference of CRP levels was detectable between the YPIII wild-type, the isogenic *hfq* mutant and the *in trans*-complemented *hfq* mutant during stationary phase at 25°C and 37°C (Fig. 6B). One the other hand, the intracellular level of Hfq, shown to be CRP-dependent in other related *Enterobacteriacea*e [87], remained unchanged in the absence of *crp* (Fig. 1C). Together, this demonstrated that CRP acts as a global sRNA regulator in *Y*. *pseudotuberculosis* YPIII in an Hfq-independent manner.

### Conclusions

Many years of research on the pathogenicity mechanisms of *Yersinia* provided great insights into the plethora of virulence and fitness factors required for the successful establishment of an infection. However, we are far from understanding the precise choreography and concerted action of transcriptional and post-transcriptional factors controlling *Yersinia* virulence. In this work, we successfully applied RNA-seq to obtain a valuable data resource that provides detailled information about the expression of the majority of genes of the enteric pathogen *Y*. *pseudotuberculosis*. The high-resolution transcriptome presented here constitutes the first single nucleotide resolution TSS map of a member of the genus *Yersinia*. In addition to creating a valuable tool for the *Yersinia* research community, it provides a framework for better analysis of identified sensory and regulatory non-coding RNAs and for studying the complex regulatory mechanisms and networks that allowed pathogenic yersiniae to become successful human pathogens. The complex expression and processing patterns show that many RNA-mediated regulations are yet to be discovered in *Yersinia*. The ongoing analysis of strain- and species-specific differences of these regulatory elements will further help us to identify how they manipulate the transcriptional output of the pathogens and contribute to phenotypic variations and/or specific adaptation to certain hosts.

We further obtained a detailed snapshot of the abundance of coding and non-coding transcripts and their sites of initiation taken at different virulence-relevant conditions. The comparative analysis of these data revealed global reprogramming of the transcriptional units controlled by CRP when growth conditions change from moderate environmental to human body temperatures, which appear to prime the bacteria for growth in the digestive tract. This includes a large set of catabolic and energy production genes that facilitate consumption of various carbon sources, e.g. dietary fibers-derived simple sugars and uncovered the existence of a thermo-controlled ‘acetate switch’ which may allow preferential growth on acetate, a predominant endproduct of the intestinal microbiota. This observation is supported by another study of our group, which demonstrated that absence of CRP perturbs in particular the fluxes of the carbon core metabolism at the level of the pyruvate-acetyl-CoA-TCA cycle node [88].

The thermally induced expression switch of virulence-relevant metabolic genes is most likely to be mediated by CRP-dependent transcriptional regulators and small non-coding RNAs (Fig. 6C). In fact, we found that CRP affects the abundance of more than 50% of all identified sRNAs, most of which are under thermal control and induced during stationary phase. Based on our data we postulate that the transcription factor CRP acts as master regulator of non-coding RNAs in *Yersinia*. The identified CRP-dependent sRNAs are likely to contribute to virulence and host adaptation by modulating metabolic pathways, which are important for efficient host colonization by *Yersinia*. Furthermore, use of non-coding RNAs to link the nutritional status of *Yersinia* to virulence may allow the pathogen to increase its energy budget upon nutrient limitation by replacing proteinaceous regulators by regulatory RNA elements. Considering the wide range of reservoirs, including the different niches in the human body, a global switching between regulons and regulatory elements seems advantageous to rapidly and efficiently readjust gene expression during transmission between the different challenging external reservoirs and host environments.

We anticipate that a comprehensive detailed analysis of the global impact of transcriptional regulators important for bacterial fitness and virulence, coupled with the identification of post-transcriptional control mechanisms by sensory and regulatory RNA elements during growth in environmental reservoirs, transmission vectors and during the infection of animal hosts and humans will herald a new exciting era for future research. This will not only help us to elucidate the complex regulatory network adjusting gene expression to the rapidly changing demands during the process of an infection, it will also allow us to identify crucial control mechanisms which can be further exploited as potential drug targets.

## Materials and Methods

### DNA manipulation and plasmid construction

All DNA manipulations, restriction digestions, ligations and transformations were performed using standard genetic and molecular techniques [89]. The plasmids used in this work are listed in S1 Table. Oligonucleotides used for PCR and sequencing were purchased from Metabion and are also listed in S1 Table. Plasmid DNA was isolated using Qiagen plasmid preparation kits. DNA-modifying enzymes and restriction enzymes were purchased from New England Biolabs. PCRs were performed in a 100 μl volume for 29 cycles using Phusion High-Fidelity DNA polymerase (New England Biolabs). Purification of PCR products was routinely performed using the QIAquick kit (Qiagen). The constructed plasmid was sequenced by the in-house facility. To generate plasmid pBW131, the entire *hfq* gene along with 847 nt upstream region and 42 nt downstream region was amplified by PCR using primers V75 and V80 and cloned into the *Bam*HI site of pHSG575.

### Bacterial strains and RNA isolation

*Y*. *pseudotuberculosis* YPIII and the isogenic *crp* mutant YP89 were grown in LB medium to exponential phase (OD_600_ 0.5) and stationary phase (16 h) at 25°C and 37°C (S8 Fig). Total bacterial RNA was isolated by a hot phenol extraction protocol [89], DNA was digested using the TURBO DNase (Ambion), purified with phenol:chlorophorm:isopropanol and the quality was assessed using the Agilent RNA 6000 Nano Kit on the Agilent 2100 Bioanalyzer (Agilent Technologies) (S9 Fig). From 8 μg of total RNA the rRNA was depleted using MICROBExpress (Ambion). 1 μg of rRNA depleted RNA was treated with TAP for +TAP libraries (Epicentre Biotechnologies) (S1 Fig).

### Strand-specific library preparation and Illumina sequencing

Strand-specific RNA-seq cDNA library preparation and barcode introduction was based on RNA adapter ligation as described previously [90], omitting double strand specific nuclease normalization (DSN). In brief, the rRNA-depleted +/-TAP treated RNA was fragmented by sonication to a median size of 200 nt. The fragments were 5’-phosphorylated and ligated to 3’- and 5’-RNA-adapter oligonucleotides (S1 Fig). After reverse transcription, cDNA libraries were PCR amplified (15 cycles). Quality of the libraries was validated using Agilent 2100 Bioanalyzer (Agilent Technologies) following the manufacturer’s instruction. Cluster generation was performed using the Illumina cluster station. Single-end sequencing on the HiSeq2500 and Genome Analyzer IIx followed a standard protocol. The fluorescent images were processed to sequences and transformed to FastQ format using the Genome Analyzer Pipeline Analysis software 1.8.2 (Illumina). The sequence output was controlled for general quality features, sequencing adapter clipping and demultiplexing using the fastq-mcf and fastq-multx tool of ea-utils [91].

### Read mapping, bioinformatics and statistics

Quality of the sequencing output was analyzed using FastQC (Babraham Bioinformatics). All sequenced libraries were mapped to the YPIII genome (NC_010465) and the pYV plasmid (accessions NC_006153) using Bowtie2 (version 2.1.0) [92] with default parameters. After read mapping, SAMtools [93] was employed to filter the resulting bam files for uniquely mapped reads (both strands), which were the basis for downstream analyses. In summary, between 36–95% and 63–97% of the cDNA reads in the +TAP and-TAP libraries could be mapped to the YPIII sequence, of which 3–22% of +TAP reads and 2–23% of-TAP reads mapped uniquely to the genome.

### Identification of transcription start sites (TSSs)

In a preliminary step, sample libraries were normalized to million uniquely mapped reads and for every base the coverage and the number of reads starting at the respective position were calculated. Then, biological replicates were combined/merged by averaging coverage and read starts data. For detection of TSSs that are associated with mRNAs an adapted method of Schlüter *et al*. [94] was applied on YPIII +TAP and YP89 +TAP libraries. Initially, the genome was scanned for potential TSSs. A TSS was defined as the starting position of at least 10 cDNA reads which map to the same genomic position at their 5’-end. Moreover, the read coverage of these reads had to be at least 2-fold higher than the coverage of overlapping reads. In the next step, the potential TSSs were extended in downstream direction to transcripts of continuous coverage and classified according to the minimal transcriptional unit (MTU) model [94]. The MTU model extends the given annotation of protein coding genes by virtual 5’- and 3’-UTRs of lengths 54 nt and 20 nt for genes where 5’-UTRs are not annotated. Transcripts were retained if they could be assigned to one of three classes: mTSS (TSS of messenger RNA), lmTSS (TSS of leaderless mRNAs), rmTSS (TSS of mRNAs to be reannotated). A TSS was associated with an mRNA and classified as mTSS, if the TSS transcript started upstream of the corresponding gene and overlapped with its MTU. lmTSS represent the TSS of leaderless transcripts. In this case the 5’-UTR of the transcript is <10 nt. In some cases TSS candidates could not be identified upstream of genes, but in close proximity downstream to the start codon. TSS transcripts with a maximal distance of 70 nt were classified as rmTSS and indicate genes, which need to be reannotated. Following classification, adjacent TSS with distance ≤ 2 nt were clustered and the TSS with the highest number of read starts counted was reported as the primary TSS.

Recently, an automated approach for the detection of TSS in dRNA-seq data was proposed by Schmidtke *et al*. [95], which exploits the difference of read starts distributions between a library from untreated total RNA (-) and a library enriched for primary transcripts (+). Based on the assumption that the number of mapped reads in a defined genomic interval follows a Poisson distribution, the Skellam distribution is used to detect genomic positions, where the difference of observed read starts significantly exceeds the difference of expected read starts. These positions are then defined as TSS. We further applied TSSAR [96], an implementation of the dRNA-seq TSS detection method, on our RNA-seq data. Different to the standard dRNA-seq experimental setup, the background distribution of read starts was obtained from-TAP libraries, which are specifically depleted for cDNAs derived from fragments containing the 5’-end of primary transcripts, while the corresponding replicate of the +TAP sample represents the unbiased library (S1 Fig). p-values of TSSAR TSS predictions were added to the set of manually curated TSS and in case of matching positions (S2 Dataset). The newly identified TSSs were labelled to following conventions, x_TSS_n, where “x” indicates the YPIII genomic replicon (i.e. YPK, chromosome; YPKp, pYV virulence plasmid).

### Detection of conserved sequence motifs

To investigate potential sequence conservation at and around the newly determined TSSs, a sequence logo for the -3 to +4 neighborhoods of all 1151 TSSs was generated using the WebLogo software [60]. To compute conserved sequence motifs in the -10 and -35 promotor region we performed *de-novo* motif discovery using the MEME software [63]. As input for motif detection in the -10 region served the subsequences starting at position -15 and ending at position -3 (relative to the TSS) of all 1151 TSSs determined in this study. For the -35 region, we used the subsequences starting at position -45 and ending -25. We ran MEME in *one occurence per sequence* (OOPS) mode and searched for motifs between length three and eight for the -10 region and between length three and five for the -35 region.

### Discovery of novel non-coding RNAs

To identify expressed small regulatory RNAs, a global screen in all +TAP samples for unannotated *trans*-encoded sRNAs and *cis*-encoded antisense RNAs was performed. For this, transcripts were assembled from reads and subsequently classified. For non-coding RNA classification, TSS data were included in the YPIII annotation. In a first step, transcripts seeds, which correspond to genomic regions of minimal length of 40 nt and a continuous coverage of at least 30 reads, were identified. The resultant transcripts were extended on both ends until the coverage dropped to 10% of the transcript’s maximum coverage or if the coverage was lower than 5 reads. Finally, transcripts located in intergenic regions without overlapping UTRs were classified as *trans*-encoded sRNAs, while transcripts found on the strand opposite to a protein-coding gene were defined as *cis*-encoded antisense RNAs. Finally, all TSS and non-coding RNA candidates were inspected manually and reported if they passed this last filter. The novel non-coding RNAs were labelled according to the common convention (Ysr_n) with ongoing numbers (n).

### Differential expression analysis and identification of CRP-dependent transcripts

To detect ORFs and *trans*-encoded sRNA that are differentially expressed in YPIII and the *crp* mutant YP89, we used DESeq (version 1.12.1) [73] for all differential expression (DE) analyses following the default analysis steps described in the package’s vignette. For each comparison HTSeq in union count mode was used to generate raw read counts required by DESeq as basis for DE analysis.

### Computational prediction of CRP binding sites

In this study, a string-based prediction of conserved CRP binding site motifs was performed for *trans*-encoded sRNA which were classified as CRP-dependent by differential gene expression analysis. The CRP consensus sequence (TGTGA-6 nt-TCACA) was used to scan regions 200 nt upstream and 100 nt downstream of the sRNA 5’-end for putative CRP binding sites, allowing a maximum of two mismatches.

### Prediction of riboswitch-like elements within 5’-UTRs

Candidate mRNAs with 5’-UTRs ≥ 200 nt were screened for riboswitch-like elements (RLEs). For this purpose the entire 5’-UTR along with 50 nt of the coding region were scanned for RLEs using the RibEx Riboswitch Explorer [67].

### Data access

The high-throughput read data is deposited at the European Nucleotide Archive (ENA) with the accession no. PRJEB7268 (http://www.ebi.ac.uk/ena/data/view/PRJEB7268). A complete list of the TSSs, antisense and *trans*-encoded sRNAs is provided in the S2 and S3 Datasets. The comparative transcriptome analyses are given in S4–S6 Datasets. The annotation of global TSSs, reannotated ORFs, *trans*- and *cis*-encoded RNAs are provided as TXT files (S1–S7 Files).

### 5’ RACE and Northern blotting

Total RNA was isolated using the SV Total RNA Isolation System (Promega) as described [52]. 5’ RACE was performed using the 5’/3’ RACE Kit (Roche). For Northern blotting 10–20 μg total RNA were separated using 7 M urea/10% acrylamide/0.6 x TBE gels. The RNA was transferred onto positively charged membranes (Roche) by semi dry blotting in 1 x TBE and UV crosslinked. For radioactive labeling 20 μCi γ-ATP^32^ (Hartmann Analytic) were mixed with 10 U T4 Polynucleotide kinase (Fermentas) and 4 pmol oligonucleotide and incubated at 37°C for 1 h. The reaction was stopped by addition of STE buffer (100 mM NaCl, 10 mM Tris, pH 8, 1 mM EDTA) and the labeled oligonucleotides were purified using MicroSpin G-25 columns (GE Healthcare). The probes were mixed with 500 μg yeast tRNA (Invitrogen) and 250 μg salmon sperm DNA (Invitrogen) to reduce unspecific probe binding. Denaturation was carried out for 10 min at 95°C. Hybridization was performed in hybridization buffer (0.5 M Na_2_HPO_4_, pH 7.2, 1 mM EDTA pH 7.5, 7% [w/v] SDS) over night at 68°C. The blots were washed in washing buffer (40 mM Na_2_HPO_4_, pH 7.2, 1 mM EDTA, pH 7.5, 1% [w/v] SDS) and exposed to a Phosphorimaging screen and analyzed with a Typhoon FLA-9000 (GE Healthcare).

### Quantitative real-time RT-PCR

qRT-PCR was performed with DNA digested total RNA (1 ng/μl) using the SensiFastNoRox Kit (Bioline). Primers employed for analyzing relative gene expression are listed S1 Table. The 5S rRNA gene was used for normalization and relative gene expression was calculated as described earlier [97]. Primer efficiencies were determined experimentally using serial dilutions of genomic *Y*. *pseudotuberculosis* YPIII DNA. Primer efficiencies are: 5S rRNA: 2.22; *acnA* (YPK_2030): 2.21; *actP* (YPK_3923): 2.17; *ail* (YPK_1268): 2.15; *gntP* (YPK_0762): 2.33; *katY* (YPK_3388): 2.40; *sodC* (YPK_3445): 1.91; *uxaC* (YPK_0554): 2.15; *yopD* (pYV0054): 2.20; *yopE* (pYV0025): 2.07; *yopP/J* (pYV0098): 1.89; *yscW* (pYV0075): 2.3; *adhE* (YPK_2072): 2.03; *fliA* (YPK_2380): 1.91; *fliC* (YPK_2381): 2.03; *glnA* (YPK_4189): 2.02; *glpD* (YPK_0152): 2.22; *invA* (YPK_2429): 2.10; *pykF* (YPK_1855): 1.91; *fumC* (YPK_1985): 2.12; *glmS* (YPK_4229): 2.21; YPK_1731: 2.17; YPK_2197: 2.19; YPK_2200: 2.10.

### Western blotting

*Y*. *pseudotuberculosis* YPIII, the isogenic mutants YP89 (Δ*crp*) and YP80 (Δ*hfq*) were grown in LB medium to exponential phase (OD_600_ 0.5) and stationary phase (16 h) at 25°C and 37°C (S8 Fig). For immunological detection of Hfq, H-NS and CRP, cell extracts of equal amounts of bacteria were prepared and separated on a 15% polyacrylamide SDS gel [89]. Proteins were transferred onto an Immobilon-P membrane (Millipore) and probed with polyclonal antibodies directed against Hfq, H-NS or CRP as described [56,98].

### Purification of CRP-His_6_ and electrophoretic mobility shift assays (EMSAs)

CRP-His_6_ was purified as described [34]. For the EMSAs, the DNA fragment harboring the potential CRP binding site(s) was mixed with a control DNA fragment (*csiD* gene fragment of *E*. *coli*) in equimolar amounts. Increasing amounts of the CRP protein in 1 x binding buffer (10 mM Tris-HCl pH 7.5, 1 mM EDTA, 5 mM DTT, 5% glycerol, 10 mM NaCl, 1 mM MgCl_2_, 0.1 mg/ml BSA) containing 0.2 mM cAMP were added and the mixture was incubated at 30°C for 20 min. Subsequently, the reactions were separated on a 4% native polyacrylamide gel and the DNA fragments visualized with ethidium bromide [34].

### DNase I footprinting

For DNase I footprinting, segments of the *sR018* and *ysr212* promoters harboring the predicted CRP binding site were amplified by PCR using a digoxigenin (DIG)-labelled primer and a non-labelled primer (S1 Table). PCR fragments were purified, incubated with the purified CRP protein in the presence or absence of cAMP as described for the EMSAs. The PCR products were digested with DNase I of an appropriate dilution and the resulting products were separated and visualized as described previously [56]. The protected nucleotides were identified by comparison with a sequence ladder generated with the same DIG-labelled primer used for the amplification of the fragment by PCR.

### Detection of acetate and acetyl-CoA

For the detection of acetate and acetyl-CoA we used an enzymatic assay system (BioAssay Systems) and followed the instructions of the manufacturers.

## Supporting Information

S1 Table Bacterial strains and oligonucleotides used in this study. (DOCX)

S1 Fig Workflow of the RNA-seq approach. RNA isolation, rRNA depletion, fragmentation, RNA adapter ligation and strand-specific library preparation for Illumina sequencing is illustrated. After rRNA depletion, the RNA is treated (+TAP) or not treated with TAP (-TAP) and the consequence with respect to the RNA-seq approach is highlighted in red.
(TIF)

S2 Fig Validation of non-coding RNAs identified by RNA-seq. Selected *trans-*encoded (A-C) and antisense-encoded RNAs (C-D) identified by RNA-seq were validated by Northern blotting. Left of each section: Northern blot of the identified sRNA for the condition with the highest number of RNA-seq read counts. 10 μg of total RNA were separated on 7 M urea/12% polyacrylamide gels and detected by specific radioactive labeled probes (listed in S1 Table). The size marker is indicated on the left. Right of each section: cDNA reads of the equivalent non-coding RNA visualized in the Artemis genome browser.
(PDF)

S3 Fig Comparison of gene expression changes obtained by RNA-seq and real-time qRT-PCR. Relative gene expression changes were examined for selected genes in response to temperature (A: thermo-induced, B: thermo-repressed) or growth phase (C: induced during stationary phase, D: repressed during stationary phase). Three independent cultures of the *Y*. *pseudotuberculosis* wild-type strain YPIII were grown in LB medium to exponential (E) or stationary growth phase (S) at 25°C (25) or 37°C (37). qRT-PCR was performed in technical duplicates with DNA-free total RNA (primers are listed in S1 Table). The 5S rRNA gene was used for normalization and relative gene expression changes were calculated according to Pfaffl 2001 [97]. White bars: real-time qRT-PCR; grew bars: RNA-seq.
(TIF)

S4 Fig Temperature-dependent acetate and acetyl-CoA levels. *Y*. *pseudotuberculosis* strain YPIII was grown at 25°C or 37°C to exponential or stationary phase. Equal amounts of the bacteria were lysed and acetyl-CoA (A) and acetate (B) levels were determined enzymatically. The data represent the mean ± SEM from three independent experiments analyzed with Student’s t-test. *: P<0,05; **: P<0,01; ***: P<0,001.
(TIF)

S5 Fig Temperature-dependent reprogramming of the CRP regulon. Venn diagrams illustrating the number of (A) protein-encoding genes and (B) *trans*-encoded sRNAs differentially expressed in *Y*. *pseudotuberculosis* YPIII and the isogenic *crp* deletion strain YP89 at 25°C and 37°C. Genes which are differentially regulated by at least 4-fold (p-value ≤0.05) are included in the analysis (see also S3 and S5 Datasets). (C) CRP-dependent transcriptional regulators. Listed are genes of transcriptional regulators which were found to be differentially expressed in *Y*. *pseudotuberculosis* YPIII and the isogenic *crp* deletion mutant YP89 (S5 and S6 Datasets).
(TIF)

S6 Fig CRP-dependent regulation of non-coding RNAs in *Y. pseudotuberculosis* YPIII. Interaction of CRP with the regulatory regions of selected CRP-regulated sRNA genes. Individual DNA fragments with the predicted CRP-binding site(s) (yellow boxes; S3 Dataset) used for electrophoretic mobility shift assays are illustrated. An individual sRNA promoter fragment (*rybB*, *ysr232*) for which no CRP-binding site was predicted was included as negative control. Respective DNA fragments were incubated with increasing concentrations of CRP and 0.2 mM cAMP. As a negative control, cAMP was omitted in samples with the highest CRP concentration (right lane). The CRP-DNA complexes were separated on 4% polyacrylamide gels. The position of a specific higher molecular weight complex is marked with an asterisk. A molecular weight standard (M) was loaded, and the corresponding molecular weights are indicated. A *csiD* PCR fragment amplified from *E*. *coli* served as an internal negative control.
(TIF)

S7 Fig Analysis of the CRP binding sites in the promoter region of non-coding RNAs. The Digoxigenin-labelled fragments represent the promoter region of the *sR018* (A) and *ysr212* (B) non-coding RNAs harboring the predicted CRP-binding site incubated without CRP, with increasing amounts of purified CRP, or with the highest concentration of CRP in the absence of cAMP. The cAMP and CRP concentrations used for the assay are indicated. The predicted CRP binding sites are given in grey bars, the determined binding sites are illustrated by black bars, and the site hypersensitive to DNase I due to CRP binding is shown by a short arrow. The positions of the protected areas are given relative to the transcriptional initiation site of the non-coding RNAs.
(TIF)

S8 Fig Sampling of *Y. pseudotuberculosis* YPIII, the isogenic mutants YP89 (Δ*crp*) and YP80 (Δ*hfq*) during growth in LB medium at 25°C and 37°C. Overnight cultures of the *Y*. *pseudotuberculosis* wild-type strain YPIII and the isogenic *crp* (YP89) and *hfq* (YP80) mutants were diluted in fresh LB medium and grown at 25°C or 37°C. The broken lines indicate the growth stage in which the bacteria were harvested for RNA isolation.
(TIF)

S9 Fig Representative Bioanalyzer profile of YPIII total RNA isolated by the hot phenol method. (TIF)

S1 Dataset Mapping statistics of *Y. pseudotuberculosis* YPIII RNA-seq libraries. (XLSX)

S2 Dataset *Y. pseudotuberculosis* YPIII transcriptional start site (TSS) map and 5’-UTR repertoire. This table lists all identified TSSs (S2-1), validated TSSs (5’ RACE) (S2-2), predicted riboswitch-like elements (S2-3), leaderless and reannotated TSSs (S2-4) and all sRNAs which were classified as TSS (S2-5) in this study in separate sheets.
(XLSX)

S3 Dataset Identification and expression profiling of candidate regulatory RNAs in *Y. pseudotuberculosis* YPIII. This table lists *trans*-encoded (S3-1), and antisense RNAs (S3-2), as well as CRP binding site predictions (S3-3) in separate sheets.
(XLSX)

S4 Dataset Global gene expression changes in response to temperature and growth phase. Global gene expression profiles are listed in separate sheets containing either the entire set of annotated genes (S4-1, S4-3) or only genes which are defined as differentially expressed (S4-2, S4-4). Additionally, Venn lists of differentially expressed genes are included as separate sheets (S4-5, S4-6).
(XLSX)

S5 Dataset CRP-dependent gene expression profile during 25°C and 37°C stationary growth. Global gene expression profiles are listed in separate sheets containing either the entire set of annotated genes (S5-1, S5-3) or only genes which are defined as differentially expressed (S5-2, S5-4). Additionally, Venn lists of differentially expressed genes are included as separate sheets (S5-5, S5-6).
(XLSX)

S6 Dataset Global gene expression changes in response to temperature during YPIII and YP89 (YPIII Δ*crp*) stationary growth. Global gene expression profiles are listed in separate sheets containing either the entire set of annotated genes (S6-1, S6-3) or only genes which are defined as differentially expressed (S6-2, S6-4). Additionally, Venn lists of differentially expressed genes are included as separate sheets (S6-5, S6-6).
(XLSX)

S1 File TXT annotation file containing all TSSs identified on the chromosome (NC_010465). (TXT)

S2 File TXT annotation file containing all TSSs identified on the pYV virulence plasmid (NC_006153). (TXT)

S3 File TXT annotation file containing all reannotated ORFs located on the YPIII chromosome (NC_010465). (TXT)

S4 File TXT annotation file containing all reannotated ORFs located on the pYV virulence plasmid (NC_006153). (TXT)

S5 File TXT annotation file containing all *trans*-encoded sRNAs identified on the YPIII chromosome (NC_010465). (TXT)

S6 File TXT annotation file containing all antisense-encoded RNAs identified on the YPIII chromosome (NC_010465). (TXT)

S7 File TXT annotation file containing all antisense-encoded RNAs identified on the pYV virulence plasmid (NC_006153). (TXT)
