# Supplementary material for: Transcriptomic Profiling of Yersinia pseudotuberculosis Reveals Reprogramming of the Crp Regulon by Temperature and Uncovers Crp as a Master Regulator of Small RNAs
Source: PLoS Genet. 2015 Mar 27;11(3):e1005087. doi: 10.1371/journal.pgen.1005087 (PMC4376681; doi:10.1371/journal.pgen.1005087)
Supplement: S1 Table — (DOCX) [file pgen.1005087.s001.docx]

Table S1: Bacterial Strains and Oligonucleotides

**Bacterial Strains**

| **Bacterial Strains** | **Description** | **Source and Reference** |
| --- | --- | --- |
| YPIII | pIB1, wild type | (1) |
| YP80 | pIB1, Δ*hfq* | (2) |
| YP89 | pIB1, Δ*crp* | (2) |

**Plasmids**

| **Plasmid** | **Description** | **Source and Reference** |
| --- | --- | --- |
| pHSG575 | pSC101*,* Cm^R^ | (3) |
| pBW131 | pHSG575 *hfq,* Cm^R^ | This study |

| **Oligonucleotides** | | | |
| --- | --- | --- | --- |
| **Name** | **Sequence (5’- 3’ orientation)** | | **Description/Target Sequence** |
| ***Oligonucleotides used for cloning*** | | | |
| V75 | GCGCCGGGATCCGATATCCGTGATCCCGCTGAATC | | forward primer for complementation of *hfq*, creates BamHI site |
| V80 | GCGCCGGGATCCGATGGAGAACATAAGCAACGG | | reverse primer for complementation of *hfq*, creates BamHI site |
| ***Oligonucleotides used as probes*** | | | |
| II986 | GGGAGATTGGGCTAGCCAAGGAGGTGGTTCCTAGTATTACTTAAC | | *cyaR*; Ysr159; sR012 |
| III25 | GCAAACACAACATCACCACCACAAAGCCAAAAGCATTTCAGTACCTG | | *gcvB*; Ysr45/180; sR013 |
| II985 | TCGGTTTAGGTGGACGATAGGCACCAGTCTTAGGCATCATTCGGA | | *glmY*; sR011 |
| III9 | CAATAGGCACTCTAAAATCGGCTCTGCGTCATCCCTGGGTTTATG | | *glmZ*; Ysr148/153; sR016 |
| III7 | GGCCACATCACTGTGGCCAAATTAACATCTCTAATAGAAGGGATG | | *micA;* Ysr7/154; sR004 |
| III6 | GTTATTAAGCACTCACACTCCTTGCGGATAATGTGAGCACCAGC | | *micM*; Ysr145/157 |
| II982 | CCTACGCATCTGCGTAGGTCGGTGCAAATAAAAAACGATCTTTGATAC | | *omrA/B;* Ysr149/181; sR014 |
| III5 | CGGGACTACCGGGCTCCTCAATATGGGGACATCAAAGAAAAGCAG | | *rybB*;Ysr48; sR023 |
| II994 | GAGCAATGTCGTGCTTTTCTGACCAAAGCCAAAGACTGCCTGCCT | | *ryhB*; sR009 |
| IV878 | GACTGGGGCGGCTAATATACAGCCAAATCCGATTACGTGAAGTAAAAGG | | Ysr185; *spf* (Spot42) |
| II981 | GGCCGGGGTGAGCGAAGACTGCGCCAACACCAGGGAAAAC | | *sraH (arcZ)*/sR018; |
| IV876 | GTAGGGGTACAGCAGGGATTCTGTCAGAGCTATGTTCACGTAAATGAACG | | Ysr100 |
| V357 | CGCGGGCACTTTGTCAATAACCTCATCATTATTCCTTTACG | | Ysr103 |
| II988 | GCCCCCTGGTGTTGGCTAAAATATATTCACAGCAGACTAACTTTTTCAGC | | Ysr164 |
| V761 | GGCAAAGGTTGGAGATTGCAGGTCTATAGCCCACTAGGTAAG | | Ysr191 |
| III830 | AGCGTTCGTTACCCGTTTGCTTTGCGGCGAATCTTACGGTAA | | Ysr197 |
| II991 | CGCCCCGATTGAATAAACAATCAGGGCGACGTTCCAGTAACCATTG | | Ysr201 |
| V421 | CTTTCGCACAACGCCACGCCAGTAGCTGCGCTCAATACATCG | | Ysr204 |
| V420 | GGTTTTACGCCGTTGATTTTGTCGCAGCATACGCCAGCGACCAAC | | Ysr205 |
| V351 | GGCTACTGTATCATTGCCTACATGTCGAATTTAATTCAG | | Ysr206 |
| V418 | GATGATAACGTGCTCGCGATGGAGAGAATAGGCAGAGTAAGTAAAGACG | | Ysr210 |
| V764 | CCCTCCCCGCGATCCTCGCCTTTTCTTGTTTTTCTGTTTTTCACTG | | Ysr212 |
| V417 | GGTGATGGTGATTAAATTGAACGCGATTCATAGTGGCTTCTCTGTTTGGGC | | His leader; sR008 |
| V765 | GGAAATCTCTTTCACAGCGATTTGGGTTAGCCGTCAATCAATCGGTGC | | Ysr215 |
| V766 | CAGTAACTGTGTACCATCATCAAAATGTAAAACGGTAAGTCAGAACGGCC | | Ysr218 |
| V415 | CAGACGTTATCTGTGACTGGAAGCACTAAGTTGTTGGTTAATGGCTCTC | | Ysr219 |
| IV873 | CAAAAAAGCCCACAAGGGACGCGGTGGGCCAAGTAATCAATTTTGGGTGG | | Ysr220 |
| V353 | CGGCGTCTTTACGATCTGCCTGTTCTCTCCGTCG | | Ysr226 |
| V352 | GCTATTCGACAGATTTCTCATCTCAGTGGGC | | Ysr304 |
| V411 | GACGCGCCCTTGGCTTACGTTAACGAATCGGGGTTCGTTTGC | | Ysr93 |
| IV892 | GACCTTATAGAACTGATGGAACGTAAAAGCCTCAACCAAACACAGGTTTCACGCG | | Ysr230 |
| V768 | CGCACTCAATCTGACTTAGCTCGCTACGCCTTGGTTTGTGG | | Ysr232 |
| V409 | CCGATTCGGTCCTGAATGTTGGGGCCAACCGAAGTGAAGTTG | | Ysr240 |
| V408 | GCCAATCCGTCATTTTTTCTCCCCCTCCGATGTTATTACATAAATATT ATTACATAACCGTTATTGC | | Ysr15 |
| V407 | CGAACACAGTCCTGTTTATCAAAACGGATGATGCCGATCATCTCATCCTC | | Ysr251 |
| V355 | GTTGCTGGATATCCACATTCCAATAAAAAACAATCAAGCCG | | Ysr114 |
| V405 | CCTGTTTGCATCAACGGTGCGGTAGCTGTTCCATTCACCCGAATC | | Ysr271 |
| V403 | GGCGGATGCTTTCAGTACCAACACCGTCACCCGCGTGATTG | | Ysr276 |
| V402 | CACTGCGATGGCGCCAAATCTACTGGCTGCTGATTTCGCTGC | | Ysr283 |
|  | | |  |
| ***Oligonucleotides for electrophoretic mobility shift assay fragments*** | | | |
| V358 | CAGCAAGGCCACTCGCTC | forward primer to generate promoter fragment of *ysr206* | |
| V359 | CATGGGTTGCTCCTGTAAATATC | reverse primer to generate promoter fragment of *ysr206* | |
| V360 | GCTCATTTATGTGACTTTAATC | forward primer to generate promoter fragment of *ysr304* | |
| V361 | GCTCATTTATGTGACTTTAATC | reverse primer to generate promoter fragment of *ysr304* | |
| V362 | CGAAGATTAATCGCCCTGG | forward primer to generate promoter fragment of *ysr226* | |
| V363 | CACATATTGTTAGTTGTTGAAC | reverse primer to generate promoter fragment of *ysr226* | |
| V370 | CGTTTGCCGCCTTCCTGTAG | forward primer to generate promoter fragment of *omrA/B* | |
| V371 | GTTGATTATTCACCAATTAATACC | reverse primer to generate promoter fragment of *omrA/B* | |
| V372 | CCCTAGAGAGCATGCAGGA | forward primer to generate promoter fragment of *cyaR* | |
| V373 | GGACTATACTATCCGACTGC | reverse primer to generate promoter fragment of *cyaR* | |
| V807 | gaagttgaggtttatctggcg | forward primer to generate promoter fragment of *sraH* | |
| V808 | gcaaatgtaagcgctcttgttac | reverse primer to generate promoter fragment of *sraH* | |
| V809 | cattattaccacgttcaaaagttg | forward primer to generate promoter fragment of *ysr218* | |
| V810 | cgaacgcttctctcaggatgaac | reverse primer to generate promoter fragment of *ysr218* | |
| V815 | cggcaagatgagcgtactttac | forward primer to generate promoter fragment of *ysr212* | |
| V816 | ctatagccactcgacttgctac | reverse primer to generate promoter fragment of *ysr212* | |
| V817 | cgtattcaagttccatgagttc | forward primer to generate promoter fragment of *ysr215* | |
| V818 | ggttagccgtcaatcaatcgg | reverse primer to generate promoter fragment of *ysr215* | |
| V813 | gacgagcgggcagcaatggcg | forward primer to generate promoter fragment of *ysr191* | |
| V814 | catcatttccgagggcatagagc | reverse primer to generate promoter fragment of *ysr191* | |
| V805 | gtgatgatgattaagctattaccg | forward primer to generate promoter fragment of *ysr100* | |
| V806 | ggtagcatcctagcaagccgatcc | reverse primer to generate promoter fragment of *ysr100* | |
| V801 | GGACTTACGCTATCGCCAACG | forward primer to generate promoter fragment of *rybB* | |
| V802 | CCCGGTTAGTCTTGAACCTAAG | reverse primer to generate promoter fragment of *rybB* | |
| VI281 | CTTATGTCCCGTCACGGTAATC | forward primer to generate promoter fragment of *ysr204* | |
| VI297 | CTTTCGCACAACGCCACGCC | reverse primer to generate promoter fragment of *ysr204* | |
| V811 | CTTCATCGCGCGCTTAACCG | forward primer to generate promoter fragment of *ysr232* | |
| V812 | CCAAAATAGTCACTGTGTATATCAC | reverse primer to generate promoter fragment of *ysr232* | |
|  |  |  | |
| ***Oligonucleotides used for qRT-PCR*** | | | |
| IV116 | GACCGGCCACAACCACCG | qRT-PCR forward primer for *invA* (YPK_2429) | |
| IV117 | CCAGTTGTGGGAGTGCAGG | qRT-PCR reverse primer for *invA* (YPK_2429) | |
| IV126 | GGCCGTGAGGATGATTGGC | qRT-PCR forward primer for *katY* (YPK_3388) | |
| IV127 | CGAAGGTATGCCCTCCCGC | qRT-PCR reverse primer for *katY* (YPK_3388) | |
| IV953 | GGGGAAGGTGGAATACATTTCAG | qRT-PCR forward primer for *yopP/J* (pYV0098) | |
| IV954 | CCACATTCAGATGAGCTTCGC | qRT-PCR reverse primer for *yopP/J* (pYV0098) | |
| IV955 | GGTAGCGGAGATGGTCAGCG | qRT-PCR forward primer for *yopD* (pYV0054) | |
| IV956 | CACCAATGTTACTCATTTGCTGC | qRT-PCR reverse primer for *yopD* (pYV0054) | |
| IV959 | GGAGGGGAGCCATAAACCGG | qRT-PCR forward primer for *yopE* (pYV0025) | |
| IV960 | GTGATACTGCCACGAAGAGGG | qRT-PCR reverse primer for *yopE* (pYV0025) | |
| IV965 | GAGGTTTCAACCTGAAGTATCG | qRT-PCR forward primer for *ail* (YPK_1268) | |
| IV966 | GCCTTTCCATGACCTGCCCC | qRT-PCR reverse primer for *ail*  (YPK_1268) | |
| V36 | CTTTCTCTCTTAATGGCAAAACCC | qRT-PCR forward primer for *acnA* (YPK_2030) | |
| V37 | CTCGGTCACCACTTTAGAACCTG | qRT-PCR reverse primer for *acnA* (YPK_2030) | |
| V42 | GGTTAAAGTTCATCCCAAAGGCC | qRT-PCR forward primer for *actP* (YPK_3923) | |
| V43 | GAAGTAGCCTATAAAACCTGTGGCA | qRT-PCR reverse primer for *actP* (YPK_3923) | |
| V46 | GGCACCGTCTCTGACGCT | qRT-PCR forward primer for *adhE* (YPK_2072) | |
| V47 | CGTTTTGCACCGTCAGTTGC | qRT-PCR reverse primer for *adhE* (YPK_2072) | |
| V48 | CACGGGATGATTGATCTGTCAG | qRT-PCR forward primer for *glmS* (YPK_4229) | |
| V49 | GGACGCACAGCTGATTTGC | qRT-PCR reverse primer for *glmS* (YPK_4229) | |
| V50 | CTCAAATCACCTTATGGCATCCG | qRT-PCR forward primer for *glpD* (YPK_0152) | |
| V51 | GATCGTCAATTTTCACCTCTTTTGG | qRT-PCR reverse primer for *glpD* (YPK_0152) | |
| V83 | GTGCGTATGTCACGACCATT | qRT-PCR forward primer for *yscW* (pYV0075) | |
| V84 | TCCTCGCTCTGGAAAAGAGA | qRT-PCR reverse primer for *yscW* (pYV0075) | |
| V153 | GCTGGCATGAACGTTATGCG | qRT-PCR forward primer for *pykF* (YPK_1855) | |
| V154 | CAGTTTCATGGTCCGGATTTCAGG | qRT-PCR reverse primer for *pykF* (YPK_1855) | |
| V175 | GATGCTGAATGAGCACGAAGTG | qRT-PCR forward primer for *glnA* (YPK_4189) | |
| V177 | CGTTAATGCCTTTCCAGCCAC | qRT-PCR reverse primer for *glnA* (YPK_4189) | |
| V185 | CAGAGAACCTTCAGTAGTCTGAG | qRT-PCR forward primer for *fliC* (YPK_2381) | |
| V186 | GCACTGTTGATACGCAGACCG | qRT-PCR reverse primer for *fliC* (YPK_2381) | |
| V187 | GTTCGCCATGAAGCATTGCG | qRT-PCR forward primer for *fliA* (YPK_2380) | |
| V190 | CTGTACCGCATACGTAGTAAAC | qRT-PCR reverse primer for *fliA* (YPK_2380) | |
| V191 | CTTTACTCCAGCGCAACACTG | qRT-PCR forward primer for *sodC* (YPK_3445) | |
| V193 | CCCATTACCTTGTGGCAATG | qRT-PCR reverse primer for *sodC* (YPK_3445) | |
| V261 | GCTCAAGTGAATATGGACCTAG | qRT-PCR forward primer for *fumC* (YPK_1985) | |
| V262 | GCACGGTTTGCCAGTACCTC | qRT-PCR reverse primer for *fumC* (YPK_1985) | |
| V265 | GGTATTGGCTTAATGCTAGTGC | qRT-PCR forward primer for *gntP* (YPK_0762) | |
| V266 | GACGATTATCGCCAGTTCACC | qRT-PCR reverse primer for *gntP* (YPK_0762) | |
| V269 | GGTGACCACTATAAATGGCG | qRT-PCR forward primer for *uxaC* (YPK_0554) | |
| V270 | CAATTCAAGATGCGTCCAATGG | qRT-PCR reverse primer for *uxaC* (YPK_0554) | |
| V271 | CTGTCCGTGCTTAAGGAAAAGG | qRT-PCR forward primer for YPK_2197 | |
| V272 | GTTGCCTACTTAAGTAAACATGATCTTC | qRT-PCR reverse primer for YPK_2197 | |
| V273 | GACTAAAAATACTGCGACTAAAGTAAAAAGC | qRT-PCR forward primer for YPK_2200 | |
| V274 | GAGTGATGACACCCAAACCC | qRT-PCR reverse primer for YPK_2200 | |
| V267 | CCAAATTGTCCCAGGGCAAC | qRT-PCR forward primer for YPK_1731 | |
| V268 | CAAAAGGAACGTGGCGTTCAC | qRT-PCR reverse primer for YPK_1731 | |
| II812 | GCCTGGCGGCCATAGCGC | qRT-PCR forward primer for 5S rRNA | |
| II813 | GCCTGGCAGTGTCCTACTCT | qRT-PCR reverse primer for 5S rRNA | |
|  |  |  | |
| ***Oligonucleotides used for 5’ RACE*** | | | |
| V150 | GTAGCATCTTCGCCGCTGAAGCC | SP1 primer for *mdh* (YPK_3761) | |
| V151 | GATCAACAGCAACACCAGGCG | SP2 primer for *mdh* (YPK_3761) | |
| V159 | CTCGGACCACTTGTATACCCAAC | SP1 primer for *ldhA* (YPK_1918) | |
| V160 | CACAACGCAATGCCAGAATTTTAAC | SP2 primer for *ldhA* (YPK_1918) | |
| V163 | GTTAGTTGCCAATTGACGCCAG | SP1 primer for *focA* (YPK_2676) | |
| V164 | CTAACATTAGCCCTAAGGAGAAACA | SP2 primer for *focA* (YPK_2676) | |
| V166 | CAGAAGCAAAGTGGTTTTTGATCAC | SP1 primer for *adhE* (YPK_2072) | |
| V167 | CCAGACTCTGTTACAGCCAG | SP2 primer for *adhE* (YPK_2072) | |
| V169 | CGTTATGCACGACGGAGATATAG | SP1 primer for *glmS* (YPK_4229) | |
| V170 | GTGCCACCGTGTAAATCCTGC | SP2 primer for *glmS* (YPK_4229) | |
| V172 | GGTCATACAAGAACAAGCCGG | SP1 primer for *glpD* (YPK_0152) | |
| V173 | GAATGCGATATGCGGTGCCAG | SP2 primer for *glpD* (YPK_0152) | |
| V180 | CAGCGGCAATGCGCAAACC | SP1 primer for *aspC* (YPK_2650) | |
| V181 | GCACCGAACAGCAGTTCTTGG | SP2 primer for *aspC* (YPK_2650) | |
| V184 | CATCCTGAATAGAGTCCAGGTC | SP1 primer for *fliC* (YPK_2381) | |
| V185 | CAGAGAACCTTCAGTAGTCTGAG | SP2 primer for *fliC* (YPK_2381) | |
| V188 | CAATATCAAGAACCTGCGCGACTTC | SP1 primer for *fliA* (YPK_2380) | |
| V189 | CACTGCGAGGTGCCCAATCAC | SP2 primer for *fliA* (YPK_2380) | |
| V192 | CATGCAGATGGAAACCGTGAATTC | SP1 primer for *sodC* (YPK_3445) | |
| V193 | CCCATTACCTTGTGGCAATG | SP2 primer for *sodC* (YPK_3445) | |
|  |  |  | |
| ***Oligonucleotides used for DNA sequencing*** | |  | |
| V477 | TCACACAGGAAACAGCTATGAC | M13 reverse sequencing primer | |
|  |  |  | |
| ***Oligonucleotides used for DNase I footprinting*** | | | |
| V975 (Dig) | GCCAGCATTGTTAAGACAGAC | Digoxigenin-labeled forward primer for *sR018* (*sraH/arcZ*) | |
| V988 | GTTCAGGTATTGCGTGAGTGG | revers primer for *sR018* (*sraH/arcZ*) | |
| V981 (Dig) | CGAATAAACATATAAGTGTCCC | Digoxigenin-labeled forward primer for *ysr212* | |
| VI274 | GTGGGTAATAGCGGTAATTCG | revers primer for *ysr212* | |

**References**

1. Bolin I, Norlander I, & Wolf-Watz H (1982) Temperature-inducible outer membrane protein of *Yersinia pseudotuberculosis* and *Yersinia enterocolitica* is associated with the virulence plasmid. *Infect Immun* 37:506-512.

2. Heroven AK*, et al.* (2012) Crp induces switching of the CsrB and CsrC RNAs in *Yersinia pseudotuberculosis* and links nutritional status to virulence. *Front Cell Infect Microbiol* 2:158.

3. Takeshita S, Sato M, Tabo M, Masahashi W, & Hashimoto-Gothoh T (1987) High-copy-number and low-copy-number plasmid vectors for *lacZ* α-complemen­tation and chloramphenicol- or kanamycin resistance selection. *Gene* 61:63-74.
