# Supplementary figures and images for: Transcriptomic Profiling of Yersinia pseudotuberculosis Reveals Reprogramming of the Crp Regulon by Temperature and Uncovers Crp as a Master Regulator of Small RNAs
Source: PLoS Genet. 2015 Mar 27;11(3):e1005087. doi: 10.1371/journal.pgen.1005087 (PMC4376681; doi:10.1371/journal.pgen.1005087)

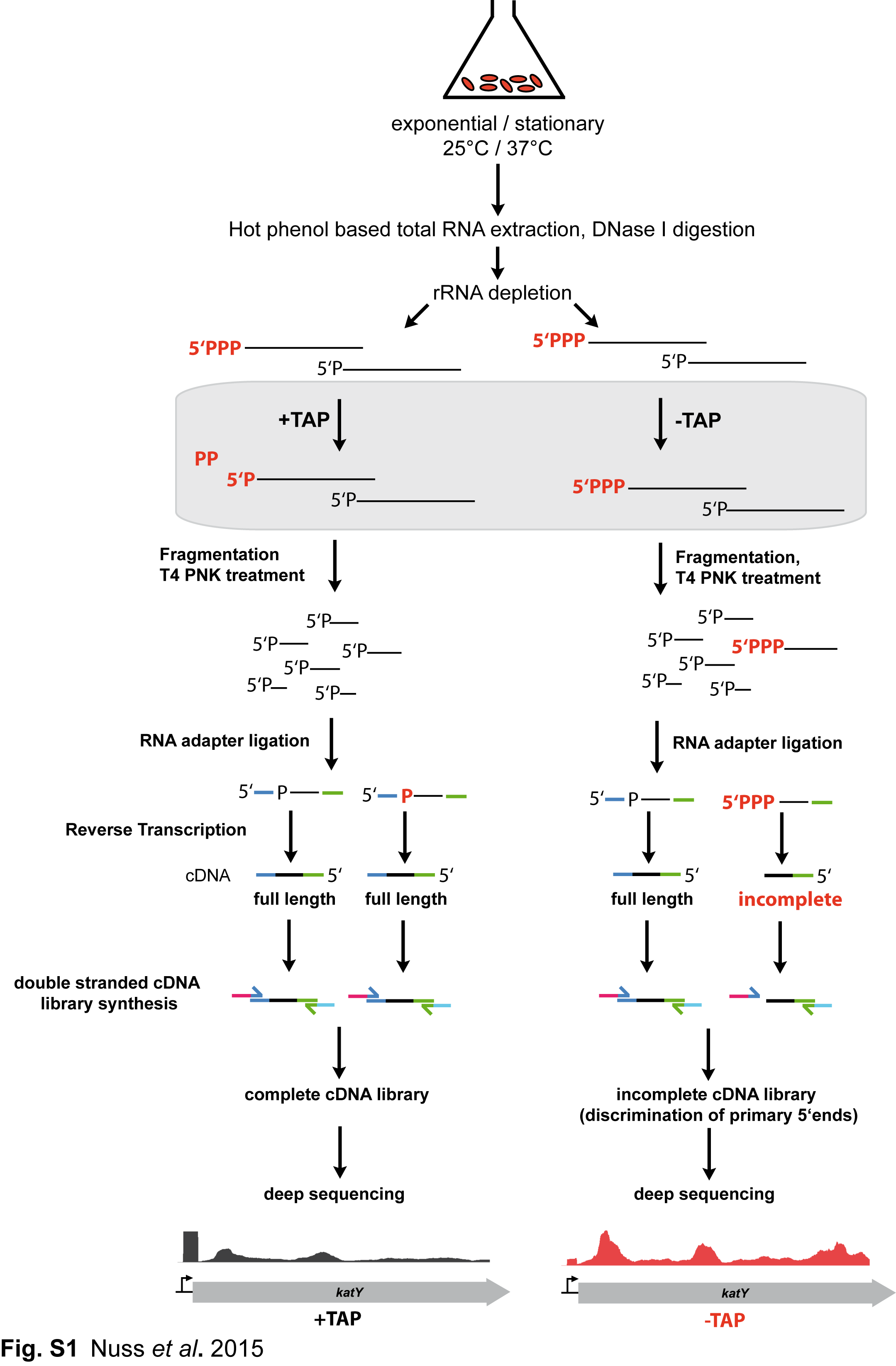

Supplement: S1 Fig — RNA isolation, rRNA depletion, fragmentation, RNA adapter ligation and strand-specific library preparation for Illumina sequencing is illustrated. After rRNA depletion, the RNA is treated (+TAP) or not treated with TAP (-TAP) and the consequence with respect to the RNA-seq approach is highlighted in red. (TIF) [file pgen.1005087.s002.tif]

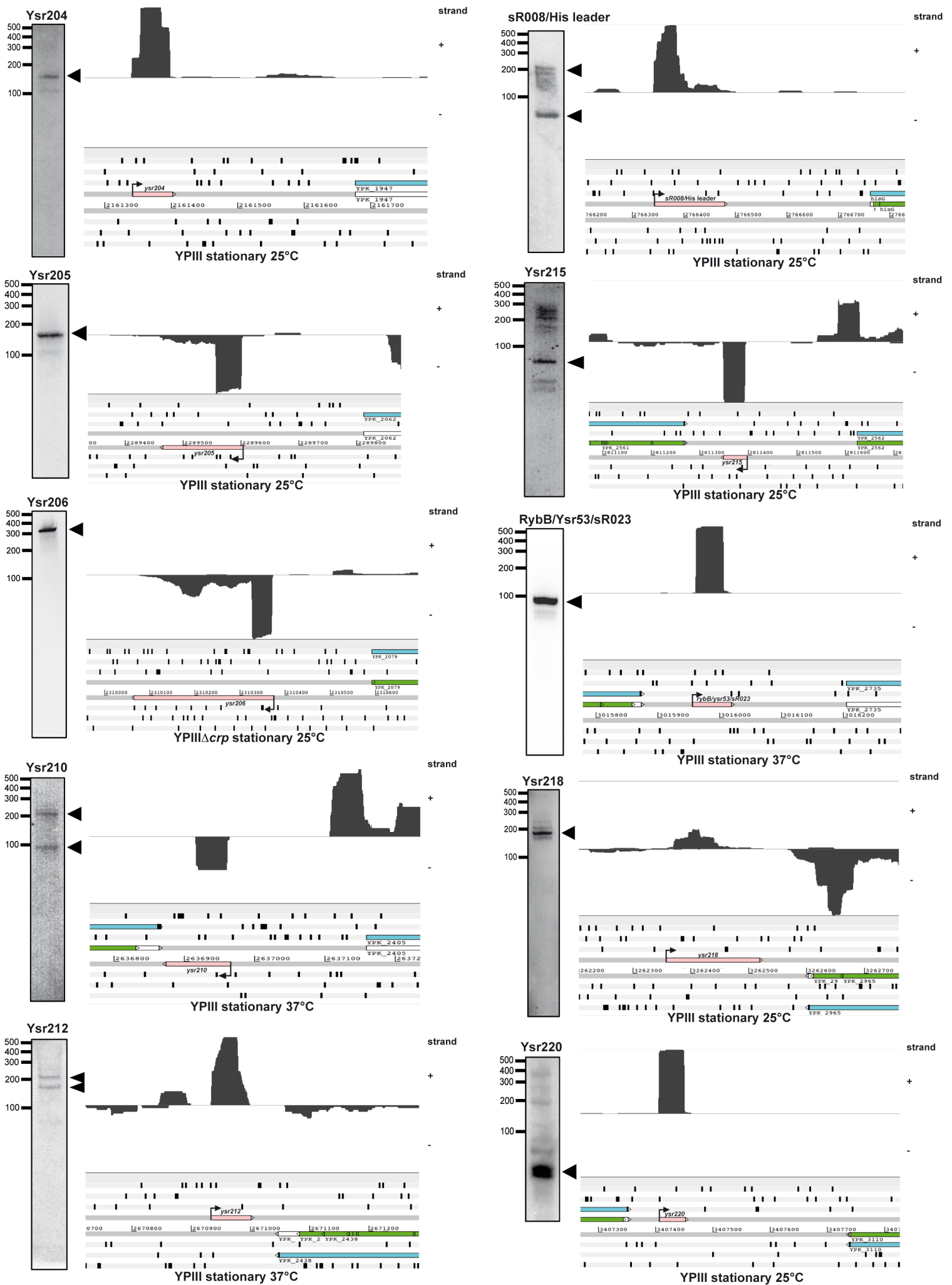

Fig. S2B Nuss *et al.* 2015

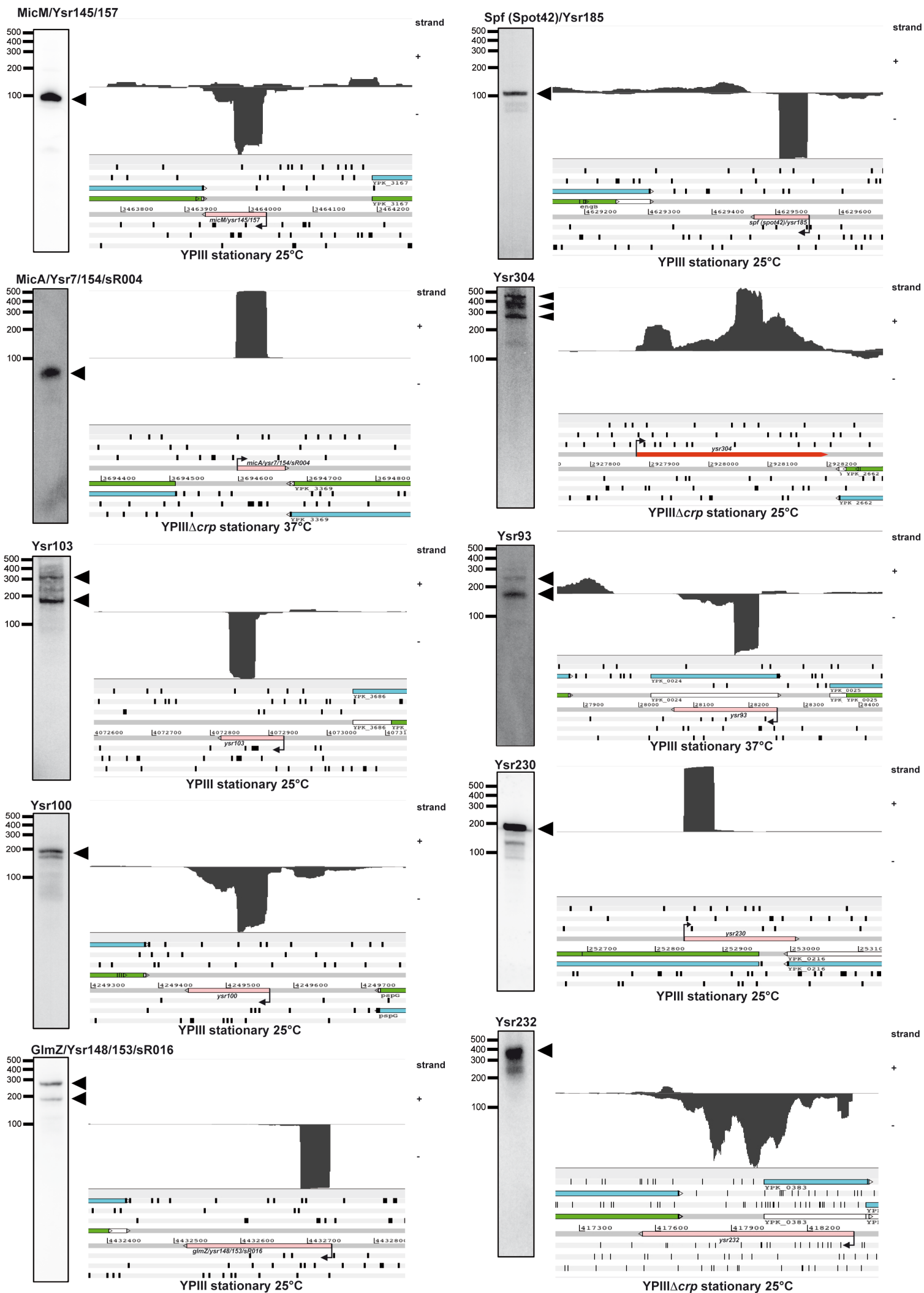

**Fig. S2C** Nuss *et al.* 2015

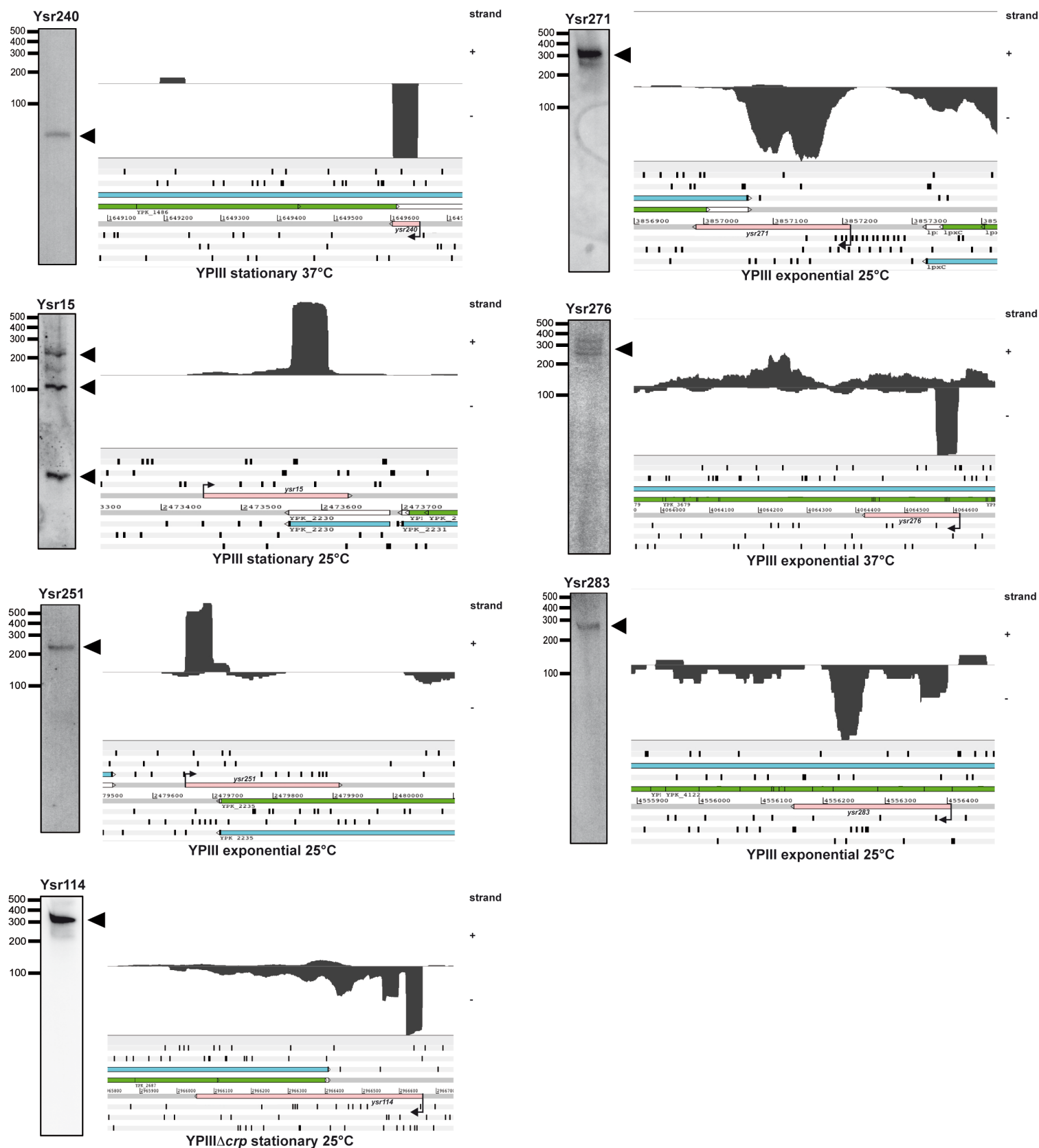

Fig. S2D Nuss *et al.* 2015

Supplement: S2 Fig — Selected trans-encoded (A-C) and antisense-encoded RNAs (C-D) identified by RNA-seq were validated by Northern blotting. Left of each section: Northern blot of the identified sRNA for the condition with the highest number of RNA-seq read counts. 10 μg of total RNA were separated on 7 M urea/12% polyacrylamide gels and detected by specific radioactive labeled probes (listed in S1 Table). The size marker is indicated on the left. Right of each section: cDNA reads of the equivalent non-coding RNA visualized in the Artemis genome browser. (PDF) [file pgen.1005087.s003.pdf]

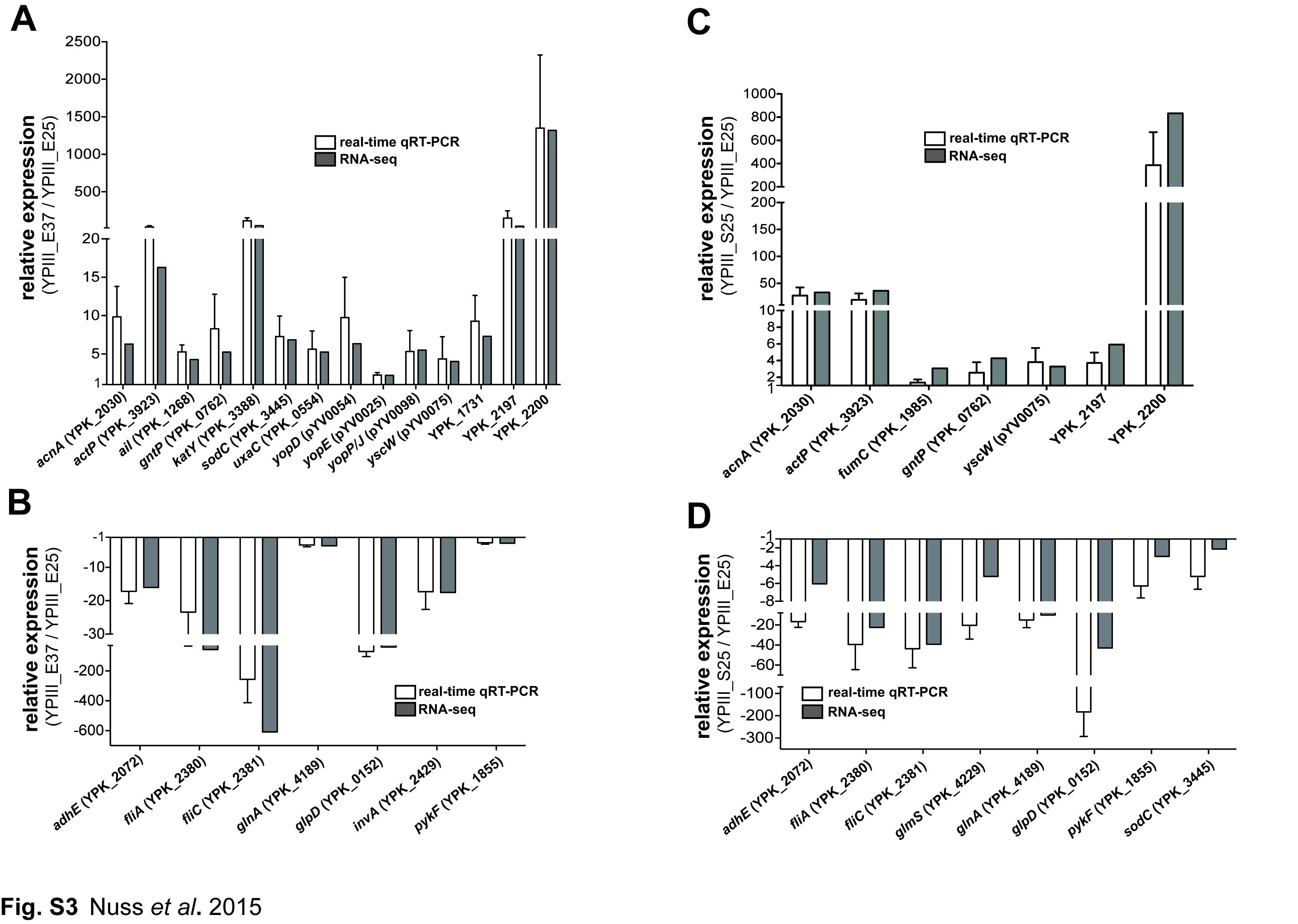

Supplement: S3 Fig — Relative gene expression changes were examined for selected genes in response to temperature (A: thermo-induced, B: thermo-repressed) or growth phase (C: induced during stationary phase, D: repressed during stationary phase). Three independent cultures of the Y. pseudotuberculosis wild-type strain YPIII were grown in LB medium to exponential (E) or stationary growth phase (S) at 25°C (25) or 37°C (37). qRT-PCR was performed in technical duplicates with DNA-free total RNA (primers are listed in S1 Table). The 5S rRNA gene was used for normalization and relative gene expression changes were calculated according to Pfaffl 2001 [97]. White bars: real-time qRT-PCR; grew bars: RNA-seq. (TIF) [file pgen.1005087.s004.tif]

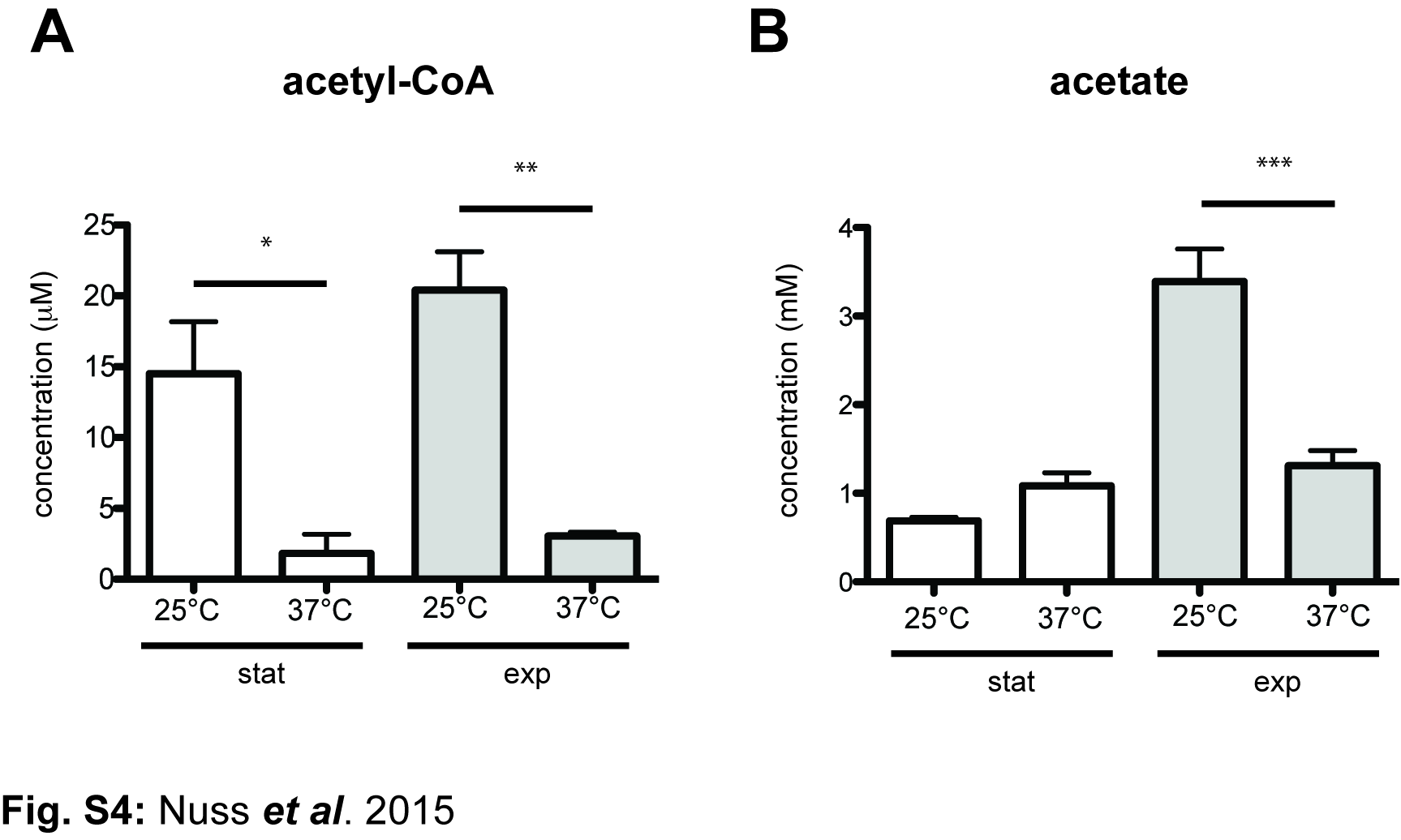

Supplement: S4 Fig — Y. pseudotuberculosis strain YPIII was grown at 25°C or 37°C to exponential or stationary phase. Equal amounts of the bacteria were lysed and acetyl-CoA (A) and acetate (B) levels were determined enzymatically. The data represent the mean ± SEM from three independent experiments analyzed with Student’s t-test. *: P<0,05; **: P<0,01; ***: P<0,001. (TIF) [file pgen.1005087.s005.tif]

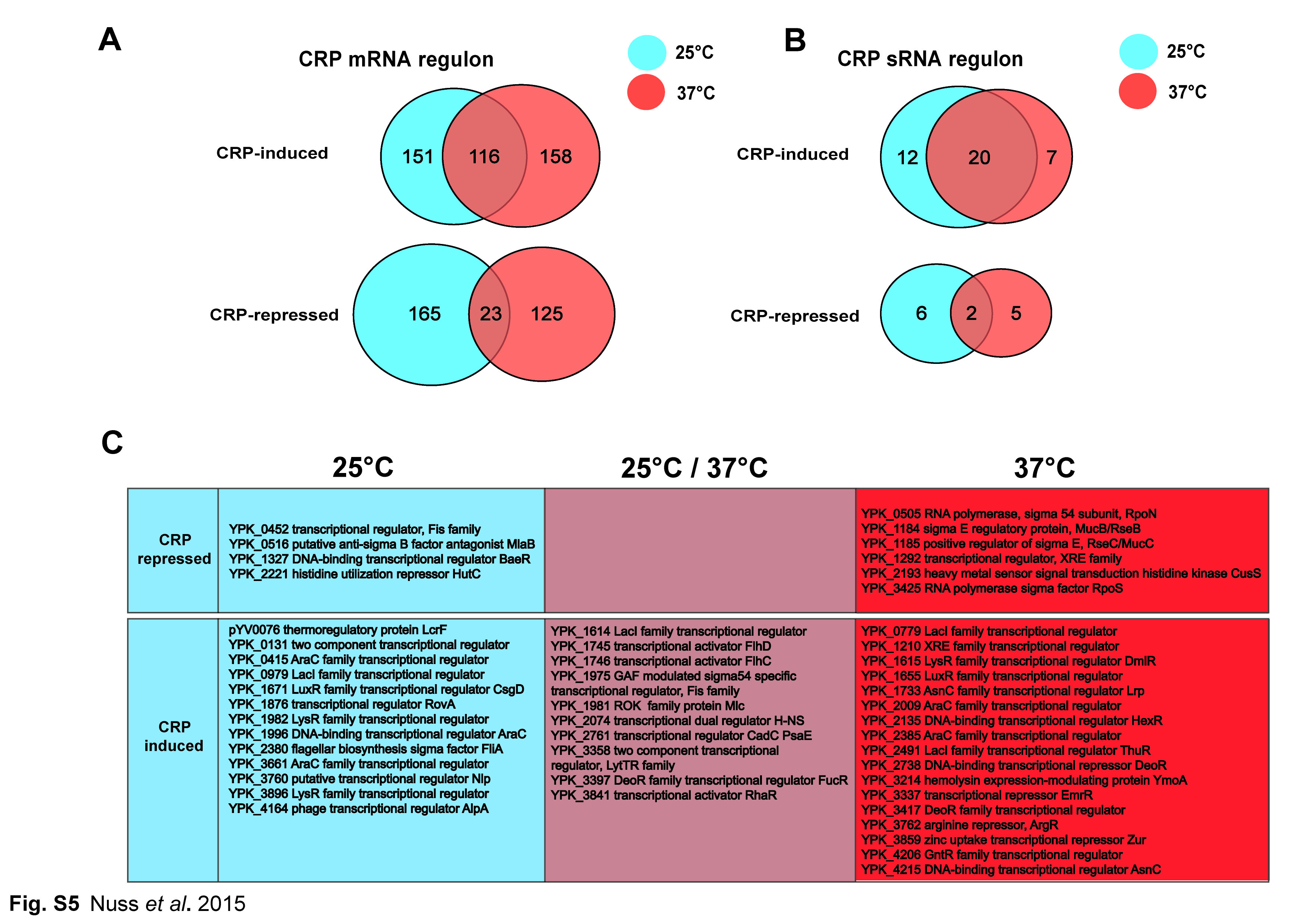

Supplement: S5 Fig — Venn diagrams illustrating the number of (A) protein-encoding genes and (B) trans-encoded sRNAs differentially expressed in Y. pseudotuberculosis YPIII and the isogenic crp deletion strain YP89 at 25°C and 37°C. Genes which are differentially regulated by at least 4-fold (p-value ≤0.05) are included in the analysis (see also S3 and S5 Datasets). (C) CRP-dependent transcriptional regulators. Listed are genes of transcriptional regulators which were found to be differentially expressed in Y. pseudotuberculosis YPIII and the isogenic crp deletion mutant YP89 (S5 and S6 Datasets). (TIF) [file pgen.1005087.s006.tif]

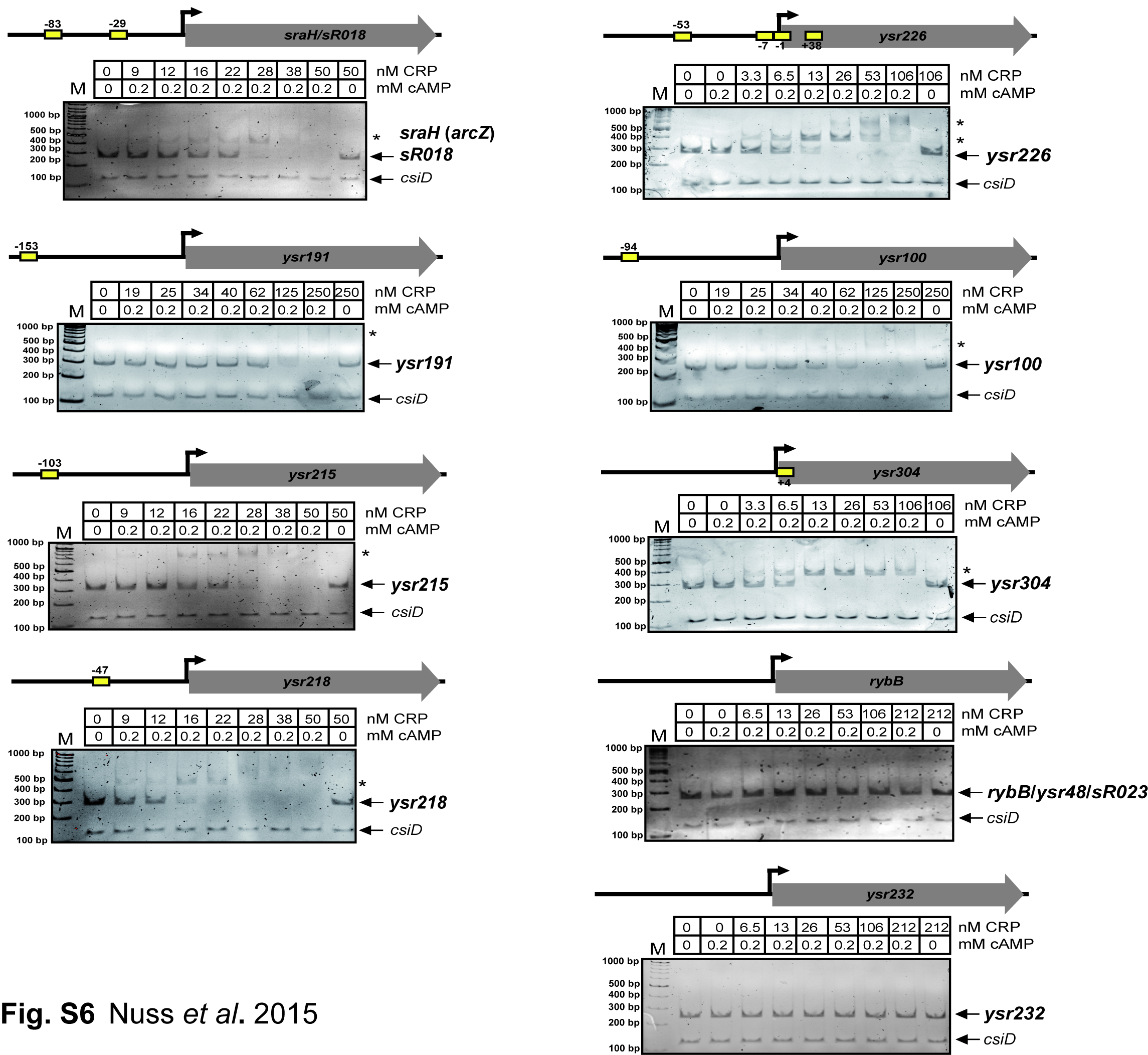

Supplement: S6 Fig — Interaction of CRP with the regulatory regions of selected CRP-regulated sRNA genes. Individual DNA fragments with the predicted CRP-binding site(s) (yellow boxes; S3 Dataset) used for electrophoretic mobility shift assays are illustrated. An individual sRNA promoter fragment (rybB, ysr232) for which no CRP-binding site was predicted was included as negative control. Respective DNA fragments were incubated with increasing concentrations of CRP and 0.2 mM cAMP. As a negative control, cAMP was omitted in samples with the highest CRP concentration (right lane). The CRP-DNA complexes were separated on 4% polyacrylamide gels. The position of a specific higher molecular weight complex is marked with an asterisk. A molecular weight standard (M) was loaded, and the corresponding molecular weights are indicated. A csiD PCR fragment amplified from E. coli served as an internal negative control. (TIF) [file pgen.1005087.s007.tif]

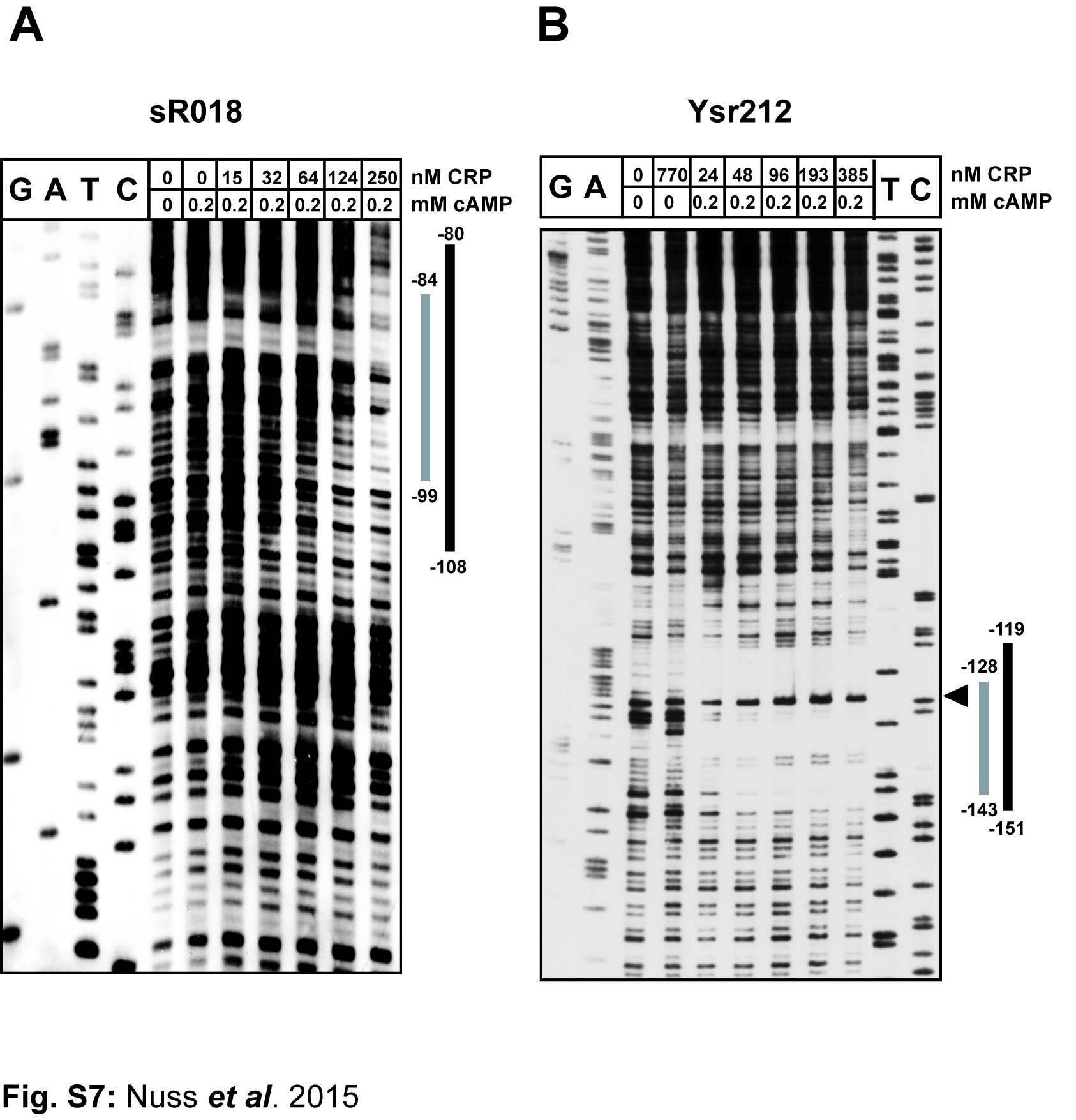

Supplement: S7 Fig — The Digoxigenin-labelled fragments represent the promoter region of the sR018 (A) and ysr212 (B) non-coding RNAs harboring the predicted CRP-binding site incubated without CRP, with increasing amounts of purified CRP, or with the highest concentration of CRP in the absence of cAMP. The cAMP and CRP concentrations used for the assay are indicated. The predicted CRP binding sites are given in grey bars, the determined binding sites are illustrated by black bars, and the site hypersensitive to DNase I due to CRP binding is shown by a short arrow. The positions of the protected areas are given relative to the transcriptional initiation site of the non-coding RNAs. (TIF) [file pgen.1005087.s008.tif]

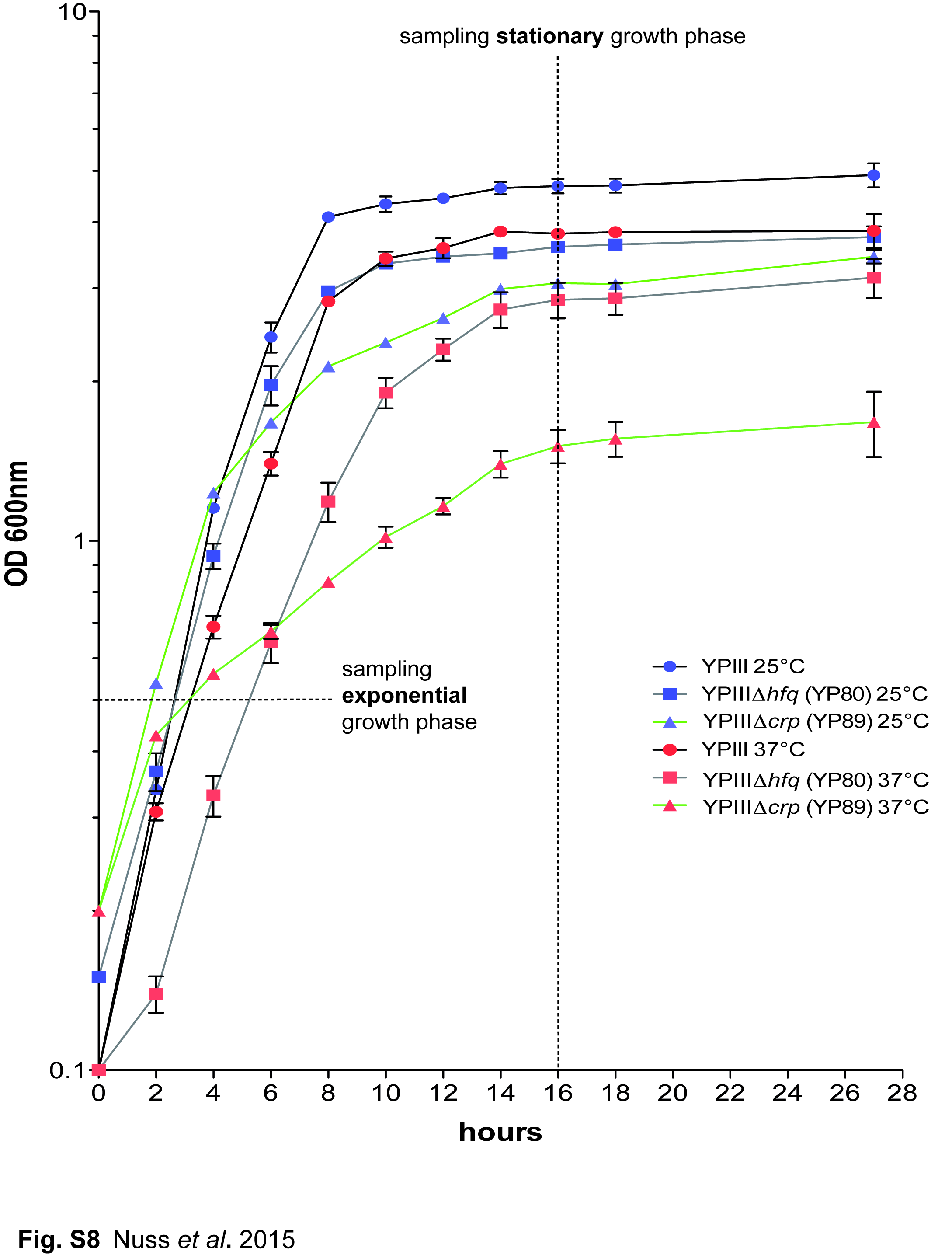

Supplement: S8 Fig — Overnight cultures of the Y. pseudotuberculosis wild-type strain YPIII and the isogenic crp (YP89) and hfq (YP80) mutants were diluted in fresh LB medium and grown at 25°C or 37°C. The broken lines indicate the growth stage in which the bacteria were harvested for RNA isolation. (TIF) [file pgen.1005087.s009.tif]

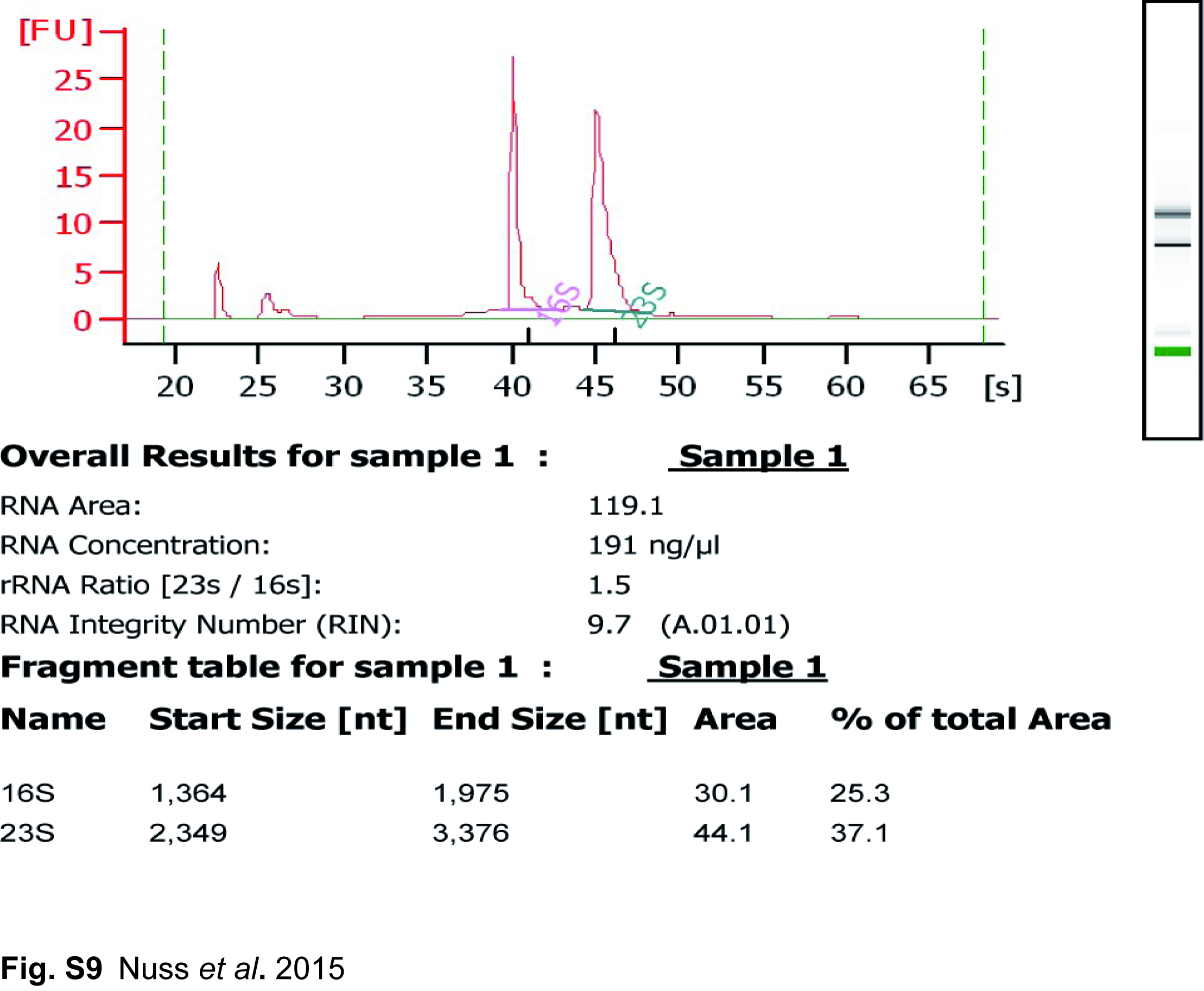

Supplement: S9 Fig — (TIF) [file pgen.1005087.s010.tif]
